# Understanding the role of the gut microbiome in gastrointestinal cancer: A review

**DOI:** 10.3389/fphar.2023.1130562

**Published:** 2023-01-24

**Authors:** Duygu Ağagündüz, Ermelinda Cocozza, Özge Cemali, Ayşe Derya Bayazıt, Maria Francesca Nanì, Ida Cerqua, Floriana Morgillo, Suna Karadeniz Saygılı, Roberto Berni Canani, Paola Amero, Raffaele Capasso

**Affiliations:** ^1^ Department of Nutrition and Dietetics, Gazi University, Emek, Ankara, Turkey; ^2^ MERCK S.P.A., Rome, Italy; ^3^ Department of Pharmacy, University of Naples “Federico II”, Naples, Italy; ^4^ Medical Oncology, Department of Precision Medicine, Università degli Studi della Campania “Luigi Vanvitelli”, Naples, Italy; ^5^ Department of Experimental Therapeutics, The University of Texas MD Anderson Cancer Center, Houston, TX, United States; ^6^ Department of Histology and Embryology, Kütahya Health Sciences University, Kütahya, Turkey; ^7^ Department of Translational Medical Science and ImmunoNutritionLab at CEINGE Biotechnologies Research Center and Task Force for Microbiome Studies, University of Naples Federico II, Naples, Italy; ^8^ Department of Agricultural Sciences, University of Naples Federico II, Portici, Italy

**Keywords:** microbiome, gastrointestinal cancer, non-coding RNAs, therapeutics, diagnosis

## Abstract

Gastrointestinal cancer represents one of the most diagnosed types of cancer. Cancer is a genetic and multifactorial disease, influenced by the host and environmental factors. It has been stated that 20% of cancer is caused by microorganisms such as *Helicobacter pylori*, hepatitis B and C virus, and human papillomavirus. In addition to these well-known microorganisms associated with cancer, it has been shown differences in the composition of the microbiota between healthy individuals and cancer patients. Some studies have suggested the existence of the selected microorganisms and their metabolites that can promote or inhibit tumorigenesis *via* some mechanisms. Recent findings have shown that gut microbiome and their metabolites can act as cancer promotors or inhibitors. It has been shown that gastrointestinal cancer can be caused by a dysregulation of the expression of non-coding RNA (ncRNA) through the gut microbiome. This review will summarize the latest reports regarding the relationship among gut microbiome, ncRNAs, and gastrointestinal cancer. The potential applications of diagnosing and cancer treatments will be discussed.

## 1 Introduction

“*Microbiome*” and “*cancer*” are the most popular topics of today’s modern age. All human surfaces and all the cavities that communicate with the outside are populated by an ecosystem of microorganisms, such as bacteria, viruses, protozoa, fungi, and archaea. This population of microbes has a complex, individual, and variable nature; its composition is influenced by the genetics of the host, dietary habits, lifestyle, and microbial exposure at birth (Zitvogel et al., 2016). An average of ten times more bacteria has been calculated than the number of cells in the human body (Guarner and Malagelada, 2003), between 500 and 1,000 different species of microorganisms. The scientific community has agreed on defining “*microbiota*” as the totality of microbial organisms present in certain environments, while the term *“microbiome”* the genetic information inherent in the microbiota itself. The microbiome presents a great variability in its composition both between different subjects and within the same subject, in different anatomical sites and tissues (Guarner et al., 2003). Individual and inter-individual differences are thought to be the underlying cause of many diseases and health problems, in gastrointestinal (GI) cancers; therefore, they play a key role in both diagnosis and treatment.

Although cancer is generally accepted as a disease caused by genetic characteristics and environmental factors, it has been reported that the effect of microorganisms on cancer could be approximately 20% of cancers human beings suffer from (De Martel et al., 2012). Digestive system cancers account for approximately one-third of cancers that result in death. At least 15%–20% of cancers are triggered by infectious agents; 20%–30% are associated with tobacco products, 30%–35% with diet, lack of physical activity, and/or energy balance disorder (obesity) (Anand et al., 2008). Targeted therapies are effective in the treatment of certain types of cancer. Many cancer types are treated by traditional chemotherapy treatments with varying efficacy and side effects. Cancer prevention is the main target, and it is reported that a better understanding of the interactions between gut microbiota, barrier function, and inflammatory responses is significant in identifying new targets in cancer therapy (Bultman, 2016; Sun and Kato, 2016).

This review aims to examine the role of the gut microbiota in the development of digestive system cancers, point out the potential factors-metabolites that can modulate this relationship, and comprehensively evaluate their potential role in diagnosis and treatment.

## 2 GI microbiome

Despite the heterogeneity found in the microbiome (Arumugam et al., 2011), based on the analysis of deoxyribonucleic acid (DNA) sequences from 39 samples belonging to subjects of six different nationalities, three main bacterial strains, which are *Bacteroides*, *Prevotella*, and *Ruminococcus* have been identified which are independent of age, sex, geographical area or diet, and based on the prevalence of one of them, each individual is cataloged in one of three enterotypes. This enterotype is linked to specific functions of the subject, such as the production of some nutrients like vitamins and the predisposition to some non-communicable diseases (NCDs) (Kau et al., 2011).

The number of microorganisms is very high in the oral cavity, where it consists mainly of *Firmicutes* and *Proteobacteria*. At the level of the esophagus, the *Firmicutes* increase even more. In the stomach involved in the secretion of hydrochloric acid in which the pH is strongly acidic (pH ≈ 2), there are a few bacteria among which the most abundant are the *Firmicutes*, *Proteobacteria*, and *Actinobacteria*. The duodenum also has a low microbial population due to the rapid transit time and the secretion of bile and pancreatic fluids that kill most of the ingested bacteria, and because of propulsive motor activity that prevents stable colonization of the lumen.

There is a progressive increase in the number of species from mouth to ileum: first, the Gram (−) and then the obligate anaerobes increase. At the level of mouth and ileum, in which the digestion and absorption of monosaccharides, amino acids, and fatty acids take place, there are *Enterococci* and *Lactobacilli*, while in the ileum and the colon (at the level of which the bile acid is absorbed) there is a myriad of bacteria, including *Ruminococcus, Staphylococcus, Streptococcus, Peptococcus, Escherichia, Eubacterium, Clostridium*, and many others are less abundant such as *Proteobacteria*, *Actinobacteria* and *Fusobacteria* (Hold et al., 2002; Hayashi et al., 2003; Eckburg et al., 2005; Hayashi et al., 2005; Wang et al., 2005; Delgado et al., 2006; Chung et al., 2016; Lu et al., 2016). The gut microbiome is the set of symbiotic microorganisms, which are bacteria, viruses, fungi, and protozoa, found in the digestive tract of mankind, about 1010-1012cells/Gram or milliliter of luminal content, 500–1,000 different species, mostly anaerobic non-sporogenous, and species that cannot be identified by conventional methods (Backhed et al., 2005; Qin et al., 2010).

Most of the species that make up the gut microbiome of the gastrointestinal tract (GIT) are included in the five main phyla in the taxonomic classification. They are listed as *Firmicutes* (60%–80%), *Bacteroidetes* (15%–25%), *Actinobacteria* (2.5%–5%), Proteobacteria (1%–10%) and *Verrucomicrobia* (0.1%–2.2%). The main bacterial phyla and species in the human digestive tract microbiota are shown in Table 1 (Tözün, 2019).

**TABLE 1:** Major bacterial phyla and species in human gut microbiota.

Phlya	Species
*Firmicutes*	*- Ruminococcus*
	*- Clostridium*
	*- Lactobacillus*
	*- Enterococcus*
*Bacteroidetes*	*- Bacteroides*
	*- Prevoetella*
	*- Xlanibacter*
*Actinobacteria*	*- Bifidobacterium*
*Proteobacteria*	*- Escherichia*
	*-* Enterobacteriaceae
*Verrucomicrobia*	*- Akkermansia muciniphila*

There is fast-growing literature examining and reporting the relationship between the bacterial composition, structure, and functional capacity of the microbiome in health and diseases. In the gut microbiome, a decrease in beneficial bacteria populations and an increase in pathogenic bacteria populations, changes in bacterial content, and deterioration of balance are defined as dysbiosis. Dysbiosis affects health negatively by preventing the regular functioning of the immune system and may play a role in the pathogenesis of some diseases (von Martels et al., 2017). Dysbiosis has been correlated with inflammatory diseases (Frank et al., 2007; Qin et al., 2010; Jostins et al., 2012), metabolic disorders (Ley et al., 2005), and tumors (Lertpiriyapong et al., 2014; Lam et al., 2017; Zitvogel et al., 2017).

## 3 Drivers of the gut microbiome composition

The lack of adequate laboratory techniques has for a long time conditioned the study of the intestinal microbiome, being the classical inadequate cultivation method since most of the flora is made up of anaerobic germs. Fortunately, the development of techniques based on the sequencing of the 16S ribosomal RNA (16S rRNA) has facilitated the identification and classification of bacteria.

The colonization of the gastrointestinal tract begins at birth with *Bacteroides* and *Bifidobacteria* in the event of a vaginal delivery. On the other hand, it mostly begins with feeding in the cesarean delivery.

In addition to the type of birth, the type of feeding is also a significant determinant of the intestinal microbiota of infants. While *Bifidobacterium* and *Lactobacillus* provide colonization in the intestinal microbiota of breastfed infants; *Enterococcus, Enterobacteria, Bacteroides, Clostridium*, and other anaerobic *Streptococcus* bacteria provide colonization in the intestinal microbiota of formula-fed infants (Groer et al., 2014). Microbiome composition and diversity may vary depending on age. The first year of life is fundamental for the establishment of the microbiome, which then variably evolves into adulthood from person to person (Gronlund et al., 1999; Harmsen et al., 2000; Palmer et al., 2007). In children between 1 and 7 years of age, there is a greater number of *Enterobacteria* than in adults with a greater abundance of general *Bifidobacterium* and *Clostridium* (Enck et al., 2009; Agans et al., 2011). The composition of the microbiota is very dynamic in the first 3 years of life and then becomes more stable and complex in adulthood (Turroni et al., 2009). In geriatric age, there is a significant variation in its composition with an increase in *Bacteroides, Escherichia coli, Streptococcus, Clostridia, Lactobacilli*, and *Bifidobacterium* decrease (Woodmansey, 2007).

The microbiome is particularly abundant in the cecum and the right colon due to the large availability of substrates and the favorable environment for bacterial growth (low transit time, ready availability of nutrients, favorable pH, *etc.*) (Savage, 1977; Bengmark, 2001). The stomach has a bacterial concentration of about 107 bacteria, therefore much lower than the distal tracts of the gastrointestinal system. The duodenum bacteria have been little studied, except for their involvement in some diseases, such as irritable bowel syndrome (IBS), in which both bacterial growth and a reduction in microbial diversity have been found (Giamarellos-Bourboulis et al., 2015). In any case, tumors of the duodenum are rare, and the relevance of bacterial involvement has not yet been investigated extensively. Besides, although the jejunum and ileum are the longest part of the gastrointestinal system and with the widest contact surface, the recovery of samples in this region remains a challenge (Wang and Yang, 2013). Molecular analyses of the 16S rRNA gene have shown that jejunum hosts a bacterial community less complex than the distal ileum and the large intestine (Wang et al., 2005); nevertheless, the knowledge on the microbial community of the small intestine is still limited both in healthy individuals and patients. On the other hand, the colon hosts one of the most populous and diverse bacterial communities that are distributed between the lumen and the mucosa (Wang et al., 2003; Eckburg et al., 2005).

Dietary patterns play a significant role in the modulation of the gut microbiome. Accumulated literature investigating the roles of nutrition in the modulation of the microbiome. Nutrition has the potential to affect gut microbiota composition and function by influencing microbial diversity, microbial taxonomy, gene expressions, and enzyme activities. Macronutrient and phytochemical content of the diet; some specific foods (probiotics, fermented foods, whole grains, oilseeds, and fruits, *etc.*) and the overall diet have the potential to modulate the gut microbiome in a short time (Beslenme and Besinler, 2017). In one of the first intervention studies examining the effects of short- and long-term dietary habits on the microbiome, it was shown that long-term dietary habits were associated with three enterotypes (*Bacteroides, Prevotella*, and *Ruminoccous*) determined through dominant groups. *Bacteroides* enterotypes were defined in diets rich in animal protein and fat, and *Prevotella* enterotypes in diets rich in complex carbohydrates. At the same time, in this study, it was shown that the change in gut microbiome started within 24 h of short-term dietary intervention but did not cause significant changes to affect enterotypes (Wu et al., 2011). In another dietary intervention study, butyrate production increased when the fiber content of the diet was increased; the fat content decreased for 2 weeks; and secondary bile acid synthesis, colonic mucosal proliferation, and inflammation have been shown to decrease (O’Keefe et al., 2015). For example, the gut microbiome of individuals who are carnivores or vegetarians differs. Changes in diet cause changes in the number and content of microorganisms in the microbiome. It was found that the ratios of *Prevotella*, *Lactobacillus*, and *Bifidobacterium* bacteria and fecal short-chain fatty acid (SCFA) levels were high in the microbiota of individuals with high adherence to the Mediterranean diet (MedDiet), and *Clostridium* species are seen to be lower (Del Chierico et al., 2014; De Filippis et al., 2016).

Interestingly, the diet influences the composition of the gut microbiome especially if the new eating habits last over time (David et al., 2014; Xu and Knight, 2015). A study suggests that low-fiber diet cause some reversible microbiota changes in mice (Sonnenburg et al., 2016), but the diversity of the microbiota is decreased if the diet continues for several generations. It is also noted that a modern diet low in fiber contributes to the loss of taxa over generations and may be responsible for the lower diversity microbiota observed in the industrialized world (Sonnenburg et al., 2016).

There is also a high interindividual and geographical diversity (De Filippo et al., 2010; Arumugam et al., 2011; Fallani et al., 2011). Nutritional habits are also reported to play a significant role in this diversity. The study of African children showed a greater presence of *Prevotella, Xylanibacter,* and *Treponema*, which possess enzymes to maximize the extraction of energy from xylans and cellulose, present in large quantities in the low-calorie but a high-fiber diet of these children (De Filippo et al., 2010). In studies conducted in adult groups, the diets of those living in rural areas were based on plant-based foods, the diversity in microbial composition was high and *Prevotella* was dominant; on the other hand, it has been found that microbial diversity is less and *Bacteroides* are dominant in those living in developed societies that consume foods rich in animal foods and processed products (Yatsunenko et al., 2012; Ou et al., 2013). All these pieces of evidence shed light on the need for well-designed studies that will evaluate the nutritional microbiota relationship from different aspects to develop correct nutritional recommendations for the regulation of microbiota.

As well as diet, even antibiotic treatments, invasive pathogens, drug-taking, or stress can disturb the balance of organisms and cause dysbiosis. It shows that antibiotic therapy in the first years of life increases the risk of inflammatory bowel disease (Shaw et al., 2010). A study showed that the microbiota is modified by the administration of antibiotic therapy (Dethlefsen et al., 2008). It has been known for a long time that excessive use of antibiotics has some negative effects on increasing antibiotic-resistant pathogens (Dethlefsen et al., 2008).

All these factors, individually or in combination, affect the intestinal microbiome, and alteration of the intestinal microbiome is related to various diseases including obesity, type 2 diabetes, atherosclerosis, cancer neurological and psychiatric diseases, inflammatory bowel diseases (IBD), and autoimmune diseases (Raoult, 2008; Sekirov et al., 2010; Holmes et al., 2011). Hence, the human microbiome including the GI tract represents a real organ adapted to protect our life and our wellbeing but is also willing to be altered in response to any factors.

## 4 Functions and metabolic clusters of the GI microbiome

The intestinal flora has a metabolic, structural, and protective role; carries out functions such as metabolizing the indigestible components of the diet, producing vitamins, contributing to the fortification of the mucosal barrier, and preventing the increase of pathogenic microorganisms. Therefore, the microbiota plays an important role in the development and regulation of the immune system. There is evidence in favor of the role of the microbiota in the development of B lymphocytes and the regulation of T helper lymphocytes. Several studies in the mouse have shown that tolerogenic dendritic cells and regulatory T cells (Tregs) are absent in germ-free mice and that both commensal bacteria and their metabolites, such as butyric acid, are necessary for the development of Tregs (Atarashi et al., 2011; Arpaia et al., 2013; Furusawa et al., 2013).

The gut microbiome also has many roles in the digestion and metabolism of nutrients. The intestinal flora, influencing the metabolism with specific enzymes, increases the extraction of energy and therefore influences the control of body weight. Resident bacteria are a fundamental line of resistance to colonization by exogenous microbes, reducing the availability of nutrients to potential pathogens (Bernet et al., 1994; Hooper et al., 1999), competing for sites of attack on the brush border of intestinal epithelial cells, and producing some antimicrobial substances (Brook, 1999; Lievin et al., 2000). Besides, among the components of the human microbiota are listed those that cause fermentation (80%) such as *Lactobacillus* and *Bifidobacteria,* and those that cause putrefaction of the remains (20%) such as *Escherichia, Bacteroides, Eubacteria*, and *Clostridium*. The fermentation of non-digestible food residues and endogenous mucus produced by the epithelium is the main source of energy in the colon. The fibers are resistant to digestion in the small intestine due to the conformation of the glycosidic bond: the enzymes of the human small intestine can only split α-type bonds, but not those of the β type. This explains why amylose, a glucose polymer with α-1,4 glycosidic bond, is digestible, while cellulose, a glucose polymer with β-1,4 glycosidic bond, is indigestible due to the lack of the specific enzyme. The distinctive feature of the carbohydrate type is the difference in hydrolysis by the enzymes of the human digestive system. Digestible carbohydrates are broken down into their monomers and absorbed from the small intestine, while non-digestible carbohydrates reach the colon. Another distinguishing feature is the use of carbohydrates reaching the colon by the colon microbiota. Carbohydrates that can be used by the microbiota can shape microbiota composition and function (Beslenme and Besinler, 2017).

In the cecum and right colon, fermentation is very intense with a high production of SCFAs (acetate, propionate, and butyrate), which generate an acid pH (range 5–6) and rapid bacterial growth. By contrast, the substrate in the left or distal colon is less available; the pH is almost neutral; the putrefactive processes become quantitatively more important, and the activity of the bacterial population is lower. Putrefaction is another anaerobic metabolism of peptides and proteins (elastin and collagen from food sources, pancreatic enzymes, exfoliated epithelial cells, and lysed bacteria, *etc.*); it also produces SCFAs but, at the same time, generates potentially toxic substances including ammonia, amines, phenols, thiols, and indoles (Macfarlane et al., 1986).

SCFAs (i.e. acetic, propionic, and butyric acids) are released because of the fermentation of some dietary components, such as dietary fiber by the colon microbiota (Smith et al., 2013). Current evidence shows that with low dietary fiber, microbial diversity is significantly reduced, and there are significant changes in microbiota composition. On the other hand, increasing dietary fiber intake increases intestinal microbial richness and/or diversity. It has also been reported to increase bacterial gene expressions for butyrate production and decrease genes required for secondary bile acid synthesis (Cotillard et al., 2013). It has been shown that long-term high fiber intake in the diet can shape the enterotype of the microbiota, especially prevotella colonization (Ou et al., 2013).

SCFAs have important functions in the physiology of the host. To illustrate, butyrate is the main source of energy for the epithelium of the colon and appears to have a protective effect against colon cancer; acetate and propionate are metabolized by the liver (propionate) or by peripheral tissues, in particular by muscles (acetate), and can play a role as modulators of glucose and cholesterol metabolism; butyrate and propionate favor the aboral transition of feces (Chen et al., 1984; Berggren et al., 1996; Wong et al., 2006). SCFAs have broad effects on many cell types, including myeloid cells with anti-inflammatory effects and regulatory T cells in the colon (Smith et al., 2013).

SCFAs can exert powerful immunomodulatory effects by suppressing the activation of the nuclear factor-kB and/or by acting on the G protein-coupled receptors as demonstrated by the acetate and increasing the expression of the Treg gene (Furusawa et al., 2013). This effect translates into some studies of an increase in immunological tolerance and protection from inflammatory or allergic diseases (Honda and Littman, 2012). On the other hand, there is also very limited evidence that this fermentation may negatively impact colon cancer (O’Keefe et al., 2015).

Unlike the other cells of the human body, which use glucose as primary energy, the colonocytes, rapidly proliferating cells, exploit butyrate for 60%–70% of their energy (Fung et al., 2012; Bode et al., 2013; Chang et al., 2014). As a fatty acid, butyrate is beta-oxidized in mitochondria (Chang et al., 2014). Interestingly, butyrate has been shown to have a higher antimutagenic effect compared to organic acids (acetic acid, lactic acid, and pyruvic acid) produced by *Lactobacillus* and *Bifidobacteria* (Chong, 2014).

According to Warburg’s hypothesis, cancer cells consume glucose (Goncalves and Martel, 2013), so the butyrate in these cells is not metabolized, accumulates in the nucleus where it inhibits histone deacetylases (HDAC) (Larrosa et al., 2006; Smith et al., 2013; Singh et al., 2014), and therefore the cell cycle progression. Moreover, the bacteria that produce SCFAs seem to affect the enterocyte cycle in the colon; in particular, butyrate inhibits cell proliferation, stimulates differentiation in epithelial neoplastic cell lines *in vitro*, and promotes the return from neoplastic to non-neoplastic phenotype (Gibson et al., 1992; Siavoshian et al., 2000). The mechanisms underlying the cancer prevention of fiber are of course not related to butyrate alone. Insoluble fiber types such as cellulose are not fermented by intestinal bacteria and accelerate intestinal transit. Decreased intestinal transit rate is thought to be effective in preventing cancer in that they reduce exposure of colon cells and exogenous carcinogens (such as heterocyclic amines from meats) (Bultman, 2016).

Finally, the colon microorganisms assisted by the presence of SCFAs also play a role in the synthesis of some vitamins (B1, B2, B3, B6, B12, pantothenic, biotin, and folic acid) and the absorption of some minerals such as calcium, magnesium, and iron (Pierro et al., 1996).

## 5 GI cancers and microbiome

### 5.1 Esophagus cancer

Esophageal cancers are the sixth leading cause of death (Mayer RJ, 2003). Normally, the number of microorganisms in the esophagus is low. It is reported that *Streptococcus viridans* is the most common in the esophageal microbiota (Yang et al., 2009). The transition from Gram (+) bacteria to Gram (−) bacteria predominance in the esophageal flora induces an important inflammatory response that triggers dysbiosis. Loss of bacterial diversity and atrophic gastritis are increasingly associated with distal esophageal cancer. Higher levels of *Campylobacter concisus* and *Campylobacter rectus* have been found in patients with premalignant Barrett’s esophagus and are associated with pathogenesis (WHO, 2020). In the scans performed on adenocarcinomas, it has been shown that the microbiota includes *Campylobacter* and some *Escherichia coli* species (Zaidi et al., 2016).

### 5.2 Gastric tumor

The involvement of *Helicobacter pylori* in gastric cancer is the best-known example of a microbial infection related to cancer in the human gastrointestinal tract. In the early 1990s, these flagellated Gram (−) bacteria were classified in group I of carcinogens by the International Agency for Cancer Research (IARC, 1994).

Several studies have shown that *Helicobacter pylori* are implicated in the onset of gastric adenocarcinoma; specifically, the deficiency of the aforementioned bacterium coincides with the increase in the incidence of this tumor form due to the consequent alterations in the physiology of the gastrointestinal tract (secretion of gastric acid, hormones, and immunocytes) and changes the composition of the microbiota since it alters the proportion of other microbial species present (Peek and Blaser, 2002). Studies have shown that changes in microbiota diversity occur during the sequence of chronic gastritis-atrophic gastritis-intestinal metaplasia-dysplasia (Aviles-Jimenez et al., 2014; Eun et al., 2014), with an increase of 21 groups, including *Peptostreptococcus stomatis, Parvimonas micra, Fusobacterium nucleatum*, and the depletion of 10 bacterial groups (Coker et al., 2018).

*Helicobacter pylori* play a role in oncogenesis at the gastric level through three mechanisms (Polk and Peek, 2010). First, *helicobacter pylori* inject two cytotoxins, VacA and CagA into the host cell, which activates oncogenic signal transduction pathways (Fuhler et al., 2009; Queiroz et al., 2012; Hoekstra et al., 2016). Secondly, it induces the production of reactive oxygen species (ROS) that activate inflammatory pathways. Finally, causing atrophic gastritis is characterized by the destruction of the parietal cells that produce acid so that there is a compensatory upregulation of gastrin that stimulates the cells to produce more acid, but also activates oncogenic signals. It is shown that *Helicobacter pylori* are responsible for chronic atrophic gastritis while it is the consequent change in the microbial flora responsible for the progression to gastric cancer. Following the reduction of gastric acidity, the carcinogenic power of some bacterial strains may increase. In addition to these, the microbiota is also populated with bacteria that reduce nitrates, it follows the formation of nitrites and carcinogenic N-nitroso compounds (Lundberg et al., 1994; Naylor and Axon, 2003; Jakszyn et al., 2006; Brawner et al., 2014).

### 5.3 Colorectal cancer

Colorectal cancer is one of the most common cancers in the world, and it is a partly hereditary tumor, partly influenced by external factors and lifestyle. The colon has the most populous bacterial community in the human body with a prevalence of *Firmicutes* and *Bacteroidetes* (Eckburg et al., 2005; Arumugam et al., 2011) and many more immune cells than other mucous membranes and lymphoid tissues. Moreover, the association between inflammation and cancer is particularly strong in colorectal cancer and indeed patients with chronic intestinal inflammation increase the risk of cancer by 2–10 times (Itzkowitz and Harpaz, 2004). Recent evidence shows that even some bacteria, such as *Bacteroides fragilis*, *Escherichia coli*, and *Peptostreptococcus anaerobius* could promote colorectal cancer by the activation of Th17 cells (Wu et al., 2009), direct DNA damage (Cuevas-Ramos et al., 2010; Arthur et al., 2012), and induction of cholesterol synthesis (Tsoi et al., 2017).

It has not yet been defined what triggers the start of the mutations responsible for the transformation of adenocarcinoma (Sears and Pardoll, 2011), but from metagenomic studies on fecal microbiota (Sobhani et al., 2011; Wang et al., 2012a; Zackular et al., 2014; Zeller et al., 2014; Feng et al., 2015; Yu et al., 2017a) and mucosal (Marchesi et al., 2011; Chen et al., 2012; Allali et al., 2015; Nakatsu et al., 2015; Flemer et al., 2017) of the patient with colorectal cancer, a dysbiosis and an abundance of *Fusobacterium*, especially *Fusobacterium nucleatum* (Castellarin et al., 2012), *Peptostreptococcus, Parvimonas*, and *Porphyromonas* (Drewes et al., 2017; Dai et al., 2018) which are the components of the oral microbiota have been noted. This suggests that carcinogenesis is associated with bacterial translocation from the mouth to the intestine (Atarashi et al., 2017).

*Fusobacterium*, in *Fusobacterium nucleatum*, is a commensal of the oral cavity, but if present in abundance in the colon-rectum, it is associated with metastasis of regional lymph nodes (Warnakulasuriya, 2009) and tumor localization in the colorectal colon (Mima et al., 2016). *Fusobacterium nucleatum* promotes colon cancer through multiple mechanisms. *Fusobacterium nucleatum* has a direct interaction with epithelial cells; it attacks and invades epithelial cells with FadA adhesion molecules that bind E-cadherin on the cell surface and activates Wnt. It is internalized and activates other inflammatory genes (Rubinstein et al., 2013). This mechanism is confirmed by the presence of high expression of the FadA gene in adenomas and colorectal tumors (Zeller et al., 2014). With the Fap2 protein, *Fusobacterium nucleatum* also binds a receptor on T lymphocytes and natural killer (NK) cells and blocks the cytotoxic activity on tumor cells (Mima et al., 2015). Moreover, the bacterium could also modify the tumor microenvironment (Kostic et al., 2013) and induce the expression of microRNA-21 (Yang et al., 2017). It is interesting to point out that the increase in *Fusobacterium nucleatum* is linked to different clinical and histological parameters, such as lower T cell infiltration (Mima et al., 2015), advanced disease status, and low survival (Flanagan et al., 2014; Mima et al., 2016). However, the difficulty is to understand the causal link: is the bacterium the cause of carcinoma or is it only the end-user of an environment altered by the tumor? There is no study confirming the cause and evidence that a transplant of specific bacteria can cause a tumor in the recipient as seen in obesity. However, there is quite convincing evidence of how extensive alterations of the bacterial flora led to the creation of a pro-oncogenic environment that favors the neoplastic transformation of the cells. This is the so-called hypothesis of the alpha bug, that is a small number of bacteria, acting as alpha subjects, would be the promoters of the carcinogenesis damaging the DNA and would favor the transformation of the remaining bacterial population of the organ concerned (Tjalsma et al., 2012).

The microbiota can also act at a distance because it can alter the intestinal barrier. The intestinal epithelium consists of a single layer of cells that separates the microbiota in the lumen from the intraepithelial lymphocytes and the cells of the innate and adaptive immune system present in the lamina propria. A thin layer of mucus produced by the goblet cells covers the epithelium of the colon and prevents direct contact of the microbes with the epithelium (Takiishi et al., 2017). A diet low in fiber can cause a condition of dysbiosis in which the butyrate-producing bacteria decrease and increase Akkermansiamuciniphilia and *Bacteroides* caccae which degrade the mucus (DeGruttola et al., 2016). All this leads to a condition of a “leaky gut”, and therefore increased permeability of the intestinal barrier. Clinical and experimental data suggest the importance of intestinal hyperpermeability in the inflammatory changes of various diseases including GI cancers (Fukui, 2016).

On the other hand, several metagenomic studies have identified significant enrichment of butyrate-producing bacteria in healthy controls compared to those affected by colorectal cancer (Itzkowitz and Harpaz, 2004) and in patients suffering from a reduction of commensal bacteria (*Bifidobacteria, lactobacillus*, *Ruminococcus*) and producing butyrate (*Lachnospiraceae* and *Faecalibacterium*) (Chen et al., 2012; Borges-Canha et al., 2015; Baxter et al., 2016; Hold, 2016). SCFAs enhance barrier functions and exert anti-inflammatory and tolerogenic effects on immune cells through mechanisms such as G-protein-coupled receptor (Gpr)-mediated sensitization of IEC inflammations and reduction of IEC oxygen concentrations and induction of hypoxia-induced factor (HIF) (Tilg et al., 2018). In this way, SCFAs have potent anti-inflammatory and anticancer effects (Bultman, 2014). Butyrate suppresses carcinogenesis through multiple mechanisms; as a histone deacetylase inhibitor, regulates cell proliferation and apoptosis (Donohoe et al., 2014), binds G proteins implicated in tumor suppression (Singh et al., 2014) and induces Treg cells, maintains the barrier function epithelial increasing the expression of tight junctions (Ploger et al., 2012) which are important for preventing inflammation. It has been found that Africans have an abundance of butyrate-producing bacteria and a reduced risk of colorectal cancer when compared to African Americans with a high risk. *Faecalibacterium prausnitzii* is the major producer of butyrate together with *Clostridium* IV and XIVa (Ou et al., 2013). *Lachnospiraceae*, another butyrate producer, is reduced in the feces of colorectal cancer patients (Baxter et al., 2016). One could imagine that the reduction of butyrate-producing bacteria and other short-chain fatty acids may contribute to the progression of colorectal cancer. It is interesting to note that *Fusobacterium* also produces butyrate but uses it as a substrate, amino acids, for example, lysine, which develop ammonia which is harmful to the intestine.

Except for all this information, the use of the microbiome in diagnosis is another question that needs to be answered. Given the spread of rectal colon cancer, biomarkers are being researched to allow the screening. Case-control cohort studies conducted in America (Zackular et al., 2014; Baxter et al., 2016; Vogtmann et al., 2016), Europe (Zeller et al., 2014; Feng et al., 2015), and China (Yu et al., 2017a) give hope that markers can be identified in the fecal microbiota as 20 genes closely associated with colorectal cancer have been found. One of these genes is that linked to the *Fusobacterium nucleatum* which also gives information on the prognosis, unfortunately negative (Flanagan et al., 2014; Mima et al., 2016; Yamaoka et al., 2018). The fecal microbiome does not correspond perfectly to the mucosal one (Flemer et al., 2017) and is more useful for identifying risk factors associated with colorectal cancer. From the diagnostic point of view, the combination of 4 markers to beat us (*Fusobacterium nucleatum, Clostridium Hathaway, Bacteriodes clarus*, and an indefinite species “m7”) turns out to be more accurate than *Fusobacterium nucleatum* alone (Liang et al., 2017). Besides, comparative studies between healthy people and patients on the oral microbiota have also identified differences that could be used as markers to diagnose esophagus cancer (Chen et al., 2015; Yu et al., 2017a; Peters et al., 2017) and pancreas (Farrell et al., 2012; Torres et al., 2015).

These data, which are likely to be used in the clinic in the future, need to be investigated with further studies. Fecal microbiota analysis showed that there is an association between colorectal cancer and increased lipopolysaccharide metabolism (LPS) which increases the activation of inflammatory pathways by TLRs whose substrates are derived from various bacterial products (LPS, PAMPs) (Zeller et al., 2014).

## 6 Potential underlying mechanisms of microbiome in GI cancers

**FIGURE 1:**
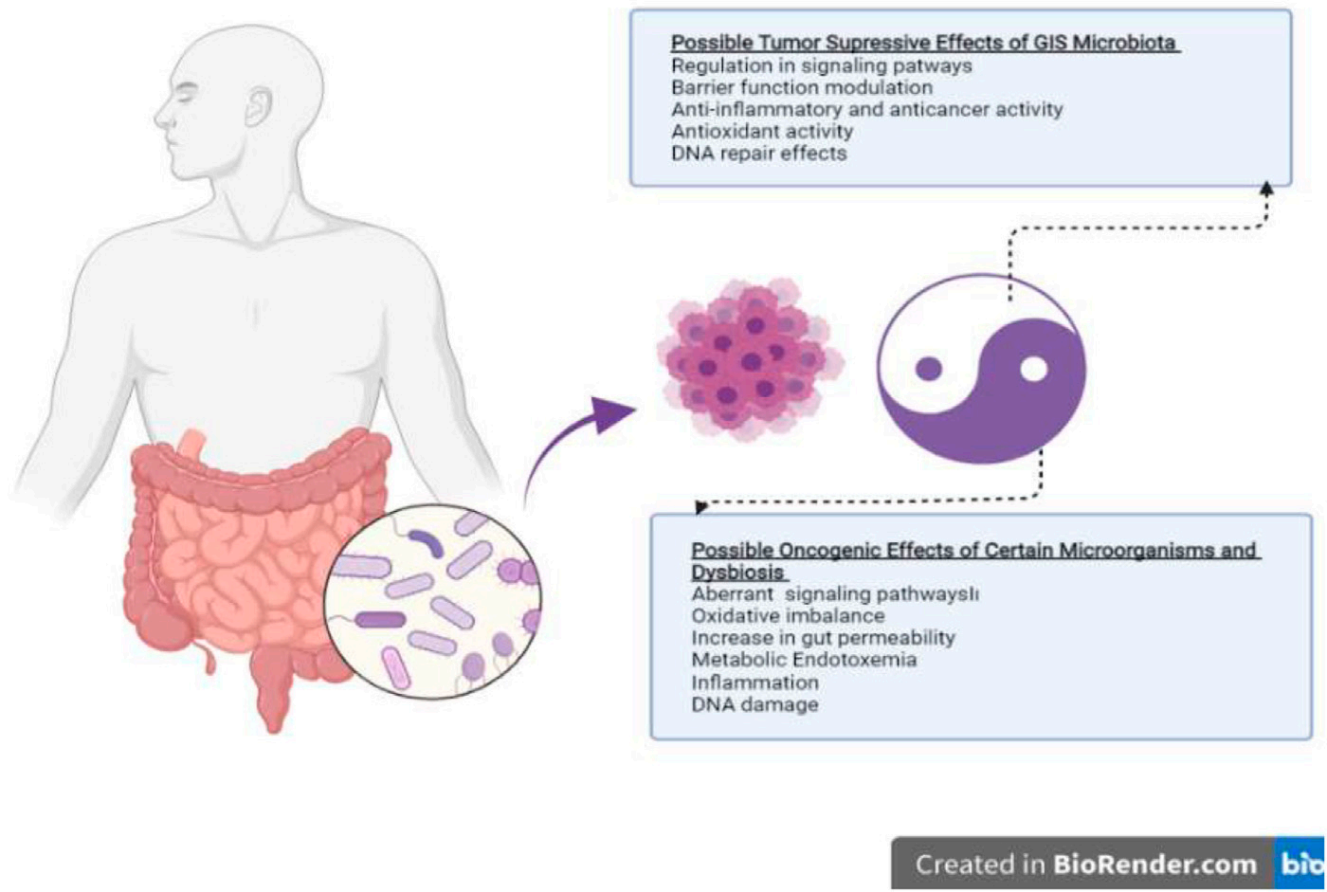
Putative mechanisms of action of the gut microbiome on tumor suppression and oncogenesis.

Possible mechanisms regarding the role of the gut microbiome in cancer development or prevention are schematized in Figure 1. There are many hypotheses about these mechanisms, and there are many factors that modulate this positive or negative effect. The relationship between cancer and microbiota has intrigued the biomedical community since the late 19th century when William Coley began treating sarcomas by injecting bacteria, the so-called “Coley’s toxin”. Subsequently, other microbial agents and their products were isolated to try to cure malignant tumors, such as bladder cancer *via Mycobacterium bovis* (Kiselyov et al., 2015) or melanoma *via* Herpes virus (Greig, 2015), or pancreatic cancer with *Listeria monocytogenes* (Le et al., 2015). However, little has yet been proven regarding the role of microbiota in the development of gastrointestinal cancers. The current evidence is carcinogenesis of certain specific bacteria found in the gut shows that it can stimulate the immune system by showing modulating effects, stimulating inflammation, and increasing cell proliferation (Abreu and Peek, 2014; Lynch and Pedersen, 2016).

Gut microbiome could modulate many systemic immune response elements such as the regulation of hematopoiesis, development of intestinal macrophages, and T cell response. The immune system participates in the initial phase of the suppression of malignant transformation, destroying the cells already infected with oncogenic viruses and recognizing the cells that express the tumor-associated antigens (TAAs) (Vesely et al., 2011). The immunogenicity of a tumor cell, and therefore its susceptibility to immunosurveillance, requires the combination of antigens, such as TAAs, and an immune system capable of responding to specific molecular patterns (DAMPs) (Kroemer et al., 2015). The intestinal mucosa signals a threat to the innate immune system, through toll-like receptors that recognize and bind specific microbial macromolecules, and this triggers a protective response by commensal bacteria, an inflammatory response to pathogenic organisms, or the trigger of apoptosis. Therefore, the commensal bacteria of the gastrointestinal tract play an active role in the development and homeostasis of the immune system.

The change in the diversity and distribution of bacteria is the most important factor that causes chronic inflammation and the onset of cancer progression. In the specific case of IBD, the mucus layer is thinner and less continuous, the tight junctions are destroyed, and the space between epithelial cells is increased. Therefore, the translocation capacity of the bacteria increases, and all this leads to the triggering of the recurrent chronic inflammatory process with the release of pro-inflammatory cytokines, Tumor Necrosis Factor (TNF), and Interferon-gamma (IFN-γ). Patients with IBD are more susceptible than others to develop colorectal cancer since chronic persistent inflammation determines an abnormal proliferation of tissue that first becomes metaplastic and then neoplastic. A few direct pieces of evidence in favor of the ability of commensal bacteria to promote tumor immunosurveillance are provided by the correlation of the growth and infiltration of major melanomas in mice with less expression in the microbiota of *Bifidobacterium* species (Sivan et al., 2015).

The increase in some tumors seems to be related to the reduction of certain microbial species, caused by modern lifestyles, which introduce ultra-processed foods (Oikonomopoulou et al., 2013) and dysbiosis by frequent antibiotic therapies. Consistent with this, an epidemiological study conducted on 125441 people demonstrated the correlation between repeated use of penicillin, cephalosporins, or macrolides with lung, prostate, and bladder cancer, and tetracycline with breast cancer (Boursi et al., 2015). Antibiotics likely favor the development of tumors by modifying the composition of the microbiota. Furthermore, the use of antibiotics during cancer therapies reduces their effectiveness. The mucosal barrier in this case has a double offense, on the part of chemotherapy and antibiotics, which can lead to the proliferation of opportunistic pathogenic bacteria that can move through the compromised epithelium and cause infection (Taur and Pamer, 2016). There is also evidence on patients confirming that treatment with Gram (+) active antibiotics is associated with shorter progression-free survival and overall survival in patients with chronic lymphatic leukemia treated with cyclophosphamide and cisplatin (Pflug et al., 2016).

Identifying microbial agents that have a causal link with the process of carcinogenesis is an important issue since it was estimated that 15.0% of new cancer cases were due to infectious agents (Plummer et al., 2016). The oncogenic mechanisms of these complex bacterial communities have not yet been fully clarified. However, there is also some evidence that some bacteria can produce toxins and metabolites that are involved in oncogenesis. Examples are CagA and VacA, the toxins produced by *Helicobacter pylori,* or deoxycholic acid, a metabolite of the genus *Clostridium* that predisposes to cancer of the colon and liver. In addition to some bacteria, there are also oncogenic viruses, such as the *Papillomaviru*s, which are positively correlated with cancer of the cervix, head-neck, bladder, and uterus (Rooney et al., 2015). Screenings of oncogenic bacteria and viruses in GI cancers may open areas of research for composing cancer control strategies.

## 7 Microbiome, diet, and GI cancers: Emerging positive and negative issues

Nutrition can prevent or increase GI cancers by modulating the microbiota. Considering the positive restoration of gut modulation, the first thing that comes to mind is undoubtedly the MedDiet. The MedDiet is among the most healthy and sustainable diet models, which is a diet model mainly based on vegetables, fruits, legumes, nuts, beans, cereals, grains, fish, and unsaturated fats such as olive oil. It has been reported that compliance with the MedDiet model positively affects the gut microbiome ecosystem and has positive roles in ensuring the immune system and metabolic balance of the human organism (Tsigalou et al., 2021). The MedDiet is based on regular consumption of monounsaturated fatty acids (MUFAs) and polyunsaturated fatty acids (PUFAs), polyphenols, and other antioxidants, high intake of prebiotic fiber and low-glycemic carbohydrates, and consumption of more plant proteins than animal proteins. Garcia-Mantrana et al. (2018) noted that a higher *Firmicutes-Bacteroidetes* ratio was associated with lower adherence to the MedDiet, whereas the greater presence of *Bacteroidetes* was associated with lower animal protein intake. Also, higher *Bifidobacteria* counts and higher total SCFAs were associated with greater consumption of plant-based nutrients such as plant proteins and polysaccharides (Garcia-Mantrana et al., 2018). Consistent with all these, it has been reported in the literature that as adherence to the MedDiet increases, mortality is lower and the frequency of gastrointestinal system diseases such as NCDs, low-grade inflammation, and Crohn’s disease decrease (Khalili et al., 2020; Tsigalou et al., 2021). Studies investigating the effects of MedDiet on GI cancers, especially colon cancer, are intense. In this context, in the European Prospective Investigation into Cancer and Nutrition (EPIC) cohort, it was found that adherence to the MedDiet delayed the onset of colorectal cancer. Although the mechanisms associated with this are not clear, it has been reported that DNA methylation of cg20674490-RUNX3 may play a mediator role in this relationship (Fasanelli et al., 2019). In another study, no negative relationship was found between MedDiet compliance and the incidence of colon, proximal colon, distal colon, and rectum cancers in the Dutch population, and this study shed light on the fact that many genomic and environmental factors can modulate this relationship (Schulpen and van den Brandt, 2020). Contrary to MedDiet, the negative effects of Western diets on the gut microbiome ecosystem have been known for a long time. The low fiber but high fat and carbohydrate content of the Western diet is the basis of dysbiosis and related GI problems (Tomasello et al., 2016). Russell et al. administered a high-protein diet for 4 weeks, which resulted in a decrease in the total number of bacteria and microorganisms that produce butyrate, which is known as a protective agent associated with lowering cancer risk, such as *Roseburia/Eubacterium* rectale (Russell et al., 2011).

Besides, the bacterial products that come from protein metabolism are thought to promote cancer and include N-nitroso compounds, ammonia, hydrogen sulfide, and polyamines (Louis et al., 2014). Roasted meat is of particular concern due to the formation of heterocyclic amines (HCAs) that are metabolized by colon bacteria in compounds that damage DNA (Huycke and Gaskins, 2004). High-fat diets significantly reduced fecal SCFAs concentration compared to low-fat diets; it is also well known to stimulate secondary bile acid secretion and increase the fecal concentration of secondary bile acids such as deoxycholic acid (DCA). Changes in the microbiota with the increase in secondary bile acids increase both intestinal permeability and endotoxin production. This situation plays an important role in the physiopathology of many chronic diseases, including cancer (Brinkworth et al., 2009). In addition, in the Western diet model, which includes frequently consumed processed foods, the intestinal barrier function may be further impaired by certain food additives, including dietary emulsifiers, and may cause significant health problems (Tilg et al., 2018).

There are also some foods and food components that are converted into onco-suppressive metabolites in GI. In the first place are the fibers and/or polysaccharides such as starch, cellulose, pectins, and gums, which reduce the risk of colorectal cancer through some mechanisms. The first mechanism is that insoluble fibers in food such as whole grains, green leafy vegetables, and dried fruits may assist in accelerating intestinal transit, therefore, reducing exposure to carcinogens that may be present. The second mechanism is that soluble fiber, contained in legumes, fresh fruit, and potatoes, sees the action of the microbiota in forming butyrate. Therefore, only soluble fibers generate butyrate, and only a long time dietary intake can bring important changes in the species of the microbiota that produce SCFAs (Sonnenburg et al., 2016; Wu et al., 2016). In addition to fibers, soy-based products such as tofu, miso, and soy milk, which contain daidzein (an isoflavone) in a form require a certain metabolism by the flora to be activated as an antioxidant. Interestingly, only 30%–40% of Westerners can metabolize daidzein in equol, while that percentage doubles in Asians (Bultman, 2016). Cruciferous vegetables are another example of bioactive compounds such as glucosinolates and their hydrolysis products including indoles and isothiocyanates (ITCs), and accumulated literature has reported that high intake of cruciferous vegetables has been associated with a lower risk of certain types of cancer including colorectal cancer (Higdon et al., 2007; Miękus et al., 2020). When these raw vegetables are cut, the glucosinolates are converted by the myrosinase (thioglucosidase) into sulforaphane (SFN) which acts as an anti-inflammatory, as an HDAC inhibitor activity (Clarke et al., 2011). However, the cruciferous are generally cooked and the heat denatures the myrosinases since that is temperature sensitive and can be inactivated upon exposure to temperatures over 60°C. However, human gut microbiota can secrete their myrosinase and can be a mediator to transform glucosinolate precursors in cruciferous vegetables to active isothiocyanates (Tian et al., 2018) and in the colon, the bacterial thioglucosidases convert the glucosinolates into isothiocyanates which exert their beneficial effects (Tian et al., 2018; Parchem et al., 2020). Finally, berries and berry constituents containing ellagic acid are metabolized to urolithin by microflora with an inhibitory function of ROS; cyclooxygenase-2 (COX-2), c-Jun (a component of activator protein-1 (AP-1)), inducible nitric oxide synthase (iNOS) expression; NF-κB and TNFα in preneoplastic tissues especially in the esophagus (Stoner et al., 2008).

The first strategies that come to mind in the prevention and treatment of GI cancers by modulation of gut microbiota or correction of dysbiosis are also biotics (Fong et al., 2020). Probiotics are identified as “live microorganisms that when administered in adequate amounts, confer a health benefit on the host” (Hill et al., 2014). The microorganisms most used as probiotics are the species from the genera *Lactobacillus, Bifidobacterium*, and other genera *Bacillus, Propionibacterium, Streptococcus, Escherichia*, and also *Saccharomyces* are also employed (O’sullivan et al., 1992; Rabah et al., 2017). They can be found and added to numerous types of products including foods and dietary supplements. Probiotic strains must be i) sufficiently characterized; ii) safe; iii) supported by at least one positive human clinical trial with accepted scientific standards and iv) alive in the product at an acceptable dose throughout shelf life (Binda et al., 2020). In the literature, it was shown that the involvement of probiotics in enhancing intestinal barrier functions, maintaining the integrity of narrow junctions, maturation of Tregs, activation of NK cells with direct elimination of bacteria, increasing mucin production from Globet cells, defensins from Paneth cells, secretion of IgA and anti-inflammatory cytokines from immune cells (Gallo et al., 2016).

Prebiotics are recently defined as “the substrates that are selectively utilized by host microorganisms conferring health benefits” (Gibson et al., 2017). Plenty of fermentable carbohydrates have been suggested to include a prebiotic effect, but the dietary prebiotics frequently reported to promote health especially intestinal benefits are the fructans (fructooligosaccharides and inulin) and galactans (galactooligosaccharides) as non-digestible oligosaccharides (Rastall and Gibson, 2015; Gibson et al., 2017). However, by the current definition, the term prebiotic not only covers oligosaccharides, but also includes other food components and metabolites (such as CLA, PUFAs, SCFAs, and dietary polyphenols), making them candidate prebiotics, additionally considering the benefits to regions outside the GI and includes various categories other than food (Gibson et al., 2017). Ingestion of prebiotics is associated with anticarcinogenic effects. Prebiotics reduce pH *via* forming SCFAs-organic acids in the colon as they are fermented and/or degraded by enzymes (β-fructanosidase β-galactosidase), which are prevalent in *Bifidobacteria* (Scott et al., 2013), thus exerting anti-inflammatory activity, stimulating Treg cells, and reducing IFN-γ (Gibson et al., 1999). Prebiotics are also able to inhibit the adhesion of pathogens to the intestinal epithelium, preventing translocation (Wang et al., 2012b; de Jesus Raposo et al., 2016; Mendis et al., 2016). Moreover, it has been shown that prebiotics increases the height of the villi, the depth of the crypts, the number of epithelial cells, and the thickness of the mucosal layer of the jejunum and the colon (Kleessen et al., 2003; de Jesus Raposo et al., 2016). Finally, another mechanism of anticancer activity is the detoxification of genotoxins such as nitrosamides and hydrogen peroxide in the gut (Wollowski et al., 2001). It plays a role in all these effects together with probiotics.

In addition to probiotics, postbiotics have recently come to the forefront due to their lower risk compared to probiotics and their modulating effect on the microbiome (Żółkiewicz et al., 2020). The International Scientific Association of Probiotics and Prebiotics (ISAPP) defined a postbiotic as a “preparation of inanimate microorganisms and/or their components that confers a health benefit on the host”. ISAPP has noted that effective postbiotics must contain inactivated microbial cells (micro and macromolecules) or cell fractions, with or without metabolites, that contribute to observed health benefits when administered in adequate amounts (Salminen et al., 2021). In addition to its health benefits, there is evidence in the literature that it supports the treatment of colorectal cancer and reduces side effects by supporting and modulating immune system function. It is reported that it does this by showing antioxidant, antiproliferative, and anti-inflammatory effects (Rad et al., 2021). However, as it is still a new emerging topic, there is a need for longer-term, large-scale randomized-controlled studies investigating the effects of postbiotics on GI cancers.

## 8 Impact of gut microbiome on anticancer therapy

It is clear in recent years that the long-term response of anticancer drugs depends on the immune response, and the microbiome can play an important role in this (Galluzzi et al., 2015). Therefore, in addition to the mechanism of carcinogenesis, the intestinal microbiota plays a role in mediating the results of anticancer therapies, giving hope for a potential improvement in treatment and reduction of toxicity through the manipulation of the microbiota. In preclinical studies, it was found that the composition of the intestinal microbiota correlates with antitumor immunity and therapeutic efficacy (Iida et al., 2013; Viaud et al., 2013; Sivan et al., 2015; Vetizou et al., 2015). For example, *Bifidobacterium breve* improves the efficacy of anti-programmed death 1 (PD-1), The cytotoxic T-lymphocyte–associated antigen 4 (CTLA-4) blockade depends on some *Bacteroides* species (Vetizou et al., 2015), and platinum-based chemotherapy requires an intact intestinal microbiota (Iida et al., 2013). Indeed, it has been shown that a massive presence of *Bifidobacterium* improves the efficacy of anti-PD-1 in reducing melanoma growth through dendritic cells that become more active in presenting melanoma antigen to T lymphocytes. In a study, microbial correlations were found in response to anti-PD-1 therapy in patients with metastatic melanoma (Peek and Crabtree, 2006). Patients who respond to therapy have an abundance of *Faelibacterium* while those who do not respond will have an abundance of other *Bacteroidales* species (Gopalakrishnan et al., 2018).

The oxaliplatin and cyclophosphamide chemotherapeutics are both less effective in tumor reduction in immunodeficient mice, in germ-free mice, or treated with broad-spectrum antibiotics (Zitvogel et al., 2016). In a recent study of patients with non-small cell renal and urothelial lung cancer, the authors found a negative correlation between antibiotic use and response to anti-PD-1 therapy (Routy et al., 2018). These results corroborate the importance of intestinal microbiota in the response to immunotherapy and that microbial destruction by antibiotics may hinder the effectiveness of immune checkpoint blockade (Jobin, 2018).

The role of intestinal microbiota has also been investigated in chemoresistance (Yu et al., 2017b), and it has been found that in the presence of *Fusobacterium nucleatum* autophagy mechanisms that confer resistance are activated. Furthermore, intestinal microbiota can predict whether a patient receiving ipilimumab will develop autoimmune colitis (Dubin et al., 2016). For instance, the toxicity of immunotherapies can be mitigated by *Bifidobacterium* (Wang et al., 2018).

## 9 GI cancer hallmarks and potential clinical applications

### 9.1 Role of non-coding RNAs and gut microbiome in GI cancers

In the last decade, the function of non-coding RNAs (ncRNAs) in cancer development has been clarified. They have a pivotal role in gene expression, cancer progression, and cell-cell communication through the involvement of extracellular vesicles (EV). ncRNAs have been classified into diverse classes based on their length, structure, and location. The important ncRNA types with well-defined roles in cancers are microRNAs (miRNA), circular RNAs (circRNA), PIWI-interacting RNAs (piRNA), and long-ncRNAs (lncRNA) (Yan and Bu, 2021). Small RNAs known as miRNAs typically have a length of 20–22 nucleotides (nt). Targeted mRNA is degraded by the RNA-induced silencing complex (RISC) when miRNAs bind to the complementary region in the target mRNA (Chang et al., 2015). piRNA, which is 24–30 nt in length, was first discovered in the *Drosophila*. It is mostly found in germline cells and interacts with members of the PIWI family of proteins to help regulate chromatin’s epigenetic state. (Tsai et al., 2020). LncRNAs and circRNAs are both longer than 200 nt, although circRNAs are ring-like whereas lncRNAs are linear. LncRNAs and circRNAs both fold into complicated second structures that allow them to interact with DNA, RNA, and proteins. They can both be created from exon, intron, intergenic region, or 5/3-untranslated sequences (Yang et al., 2018). ncRNAs can act as tumor suppressors and oncogenes in cancers and they are considered promising diagnostic and therapeutic markers. As of late numerous studies have appeared that the microbiota can influence the event and improvement of cancer by influencing the expression of ncRNA (Table 2).

**TABLE 2:** Gut microbiome-associated ncRNAs and GI cancer.

Associated gut microbiome	ncRNAs		Cancer type
	Upregulated	Downregulated	
*Helicobacter pylori*	miR99b-3p; miR-564; miR-638	miR-204-5p; miR-338-5p; miR-375; miR-548c-3p	Gastric Cancer
*Helicobacter pylori*	miR-18a-3p; miR-4286	-	Gastric Cancer
*Helicobacter pylori*	miR-223-3p	-	Gastric Cancer
*Ebstein-Barr virus*	-	miR-200a; miR-200b	Gastric Cancer
*F. nucleatum*	miR-21	-	Colorectal Cancer
*Escherichia coli*	hsa-mir-223; hsa-mir-96; hsa-mir-106a	-	Colorectal Cancer
Probiotic and pathobiont environment	LINC00355; KCNQ1OT1; LINC00491; HOTAIR		Colorectal Cancer

miRNAs play a crucial role in the relationship between the host and the microbiota in cancer. Numerous studies suggest that *Helicobacter pylori* infection may influence the host miRNA expression. Chang et al. showed that *Helicobacter pylori*-positive gastric cancer patients had considerably greater levels of miR99b-3p, miR-564, and miR-638 compared to *Helicobacter pylori*-negative patients, despite exhibiting significantly less of miR-204-5p, miR-338-5p, miR-375, and miR-548c-3p (Chang et al., 2015). MiR-18a-3p and miR-4286 levels were substantially larger in gastric cancer associated with *Helicobacter pylori*, according to research by Tsai et al. In a group of gastric cancer patients, serum expression of miR-18a-3p and miR-4286 was positively and significantly associated with *Helicobacter pylori*. Additionally, invasion, tumor stage, tumor size, and lymph node metastasis were all strongly linked with miR-18a-3p and miR-4286 (Tsai et al., 2020). Another study showed that *Helicobacter pylori* infection enhanced miR-223-3p expression, which in turn activated the NF-kB pathway and contributed to the development of gastric cancer (Yang et al., 2018). Interestingly, the *Ebstein-Barr virus* can affect cell signaling pathways, influence gene expression, cause methylation of the host genome, cause infected gastric epithelial cells to generate a tumor microenvironment, and initiate and progress gastric cancer. *Ebstein-Barr virus*-associated gastric carcinoma is a subtype of gastric cancer that has morphologic traits that are like those of cells going through the epithelial-to-mesenchymal transition (Sun et al., 2020). In one study, miR-200a and miR-200b, which are connected to the epithelial-mesenchymal transition and were assessed in gastric carcinoma, were expressed at lower levels in *Ebstein-Barr virus*-associated gastric carcinoma than in *Ebstein-Barr virus*-negative carcinoma (Shinozaki et al., 2010).

Numerous studies have demonstrated that the gut microbiome affects the expression of miRNAs and their target genes in colorectal cancer (Cougnoux et al., 2014; Yu et al., 2017b; Yang et al., 2017). According to one study, *Fusobacterium nucleatum* enhanced tumor cell miR-21 levels by turning on the TLR4-MyD88 signaling cascade, which in turn increased CRC cell growth and tumor development in mice (Yang et al., 2017). Other research has demonstrated that patients with high miR-21 and *Fusobacterium nucleatum* DNA levels consistently had a higher risk of negative outcomes. It has also been noted that *Fusobacterium nucleatum* enhances chemoresistance to CRC *via* changing autophagy in a way that is miRNA-dependent (Yu et al., 2017b).

**FIGURE 2:**
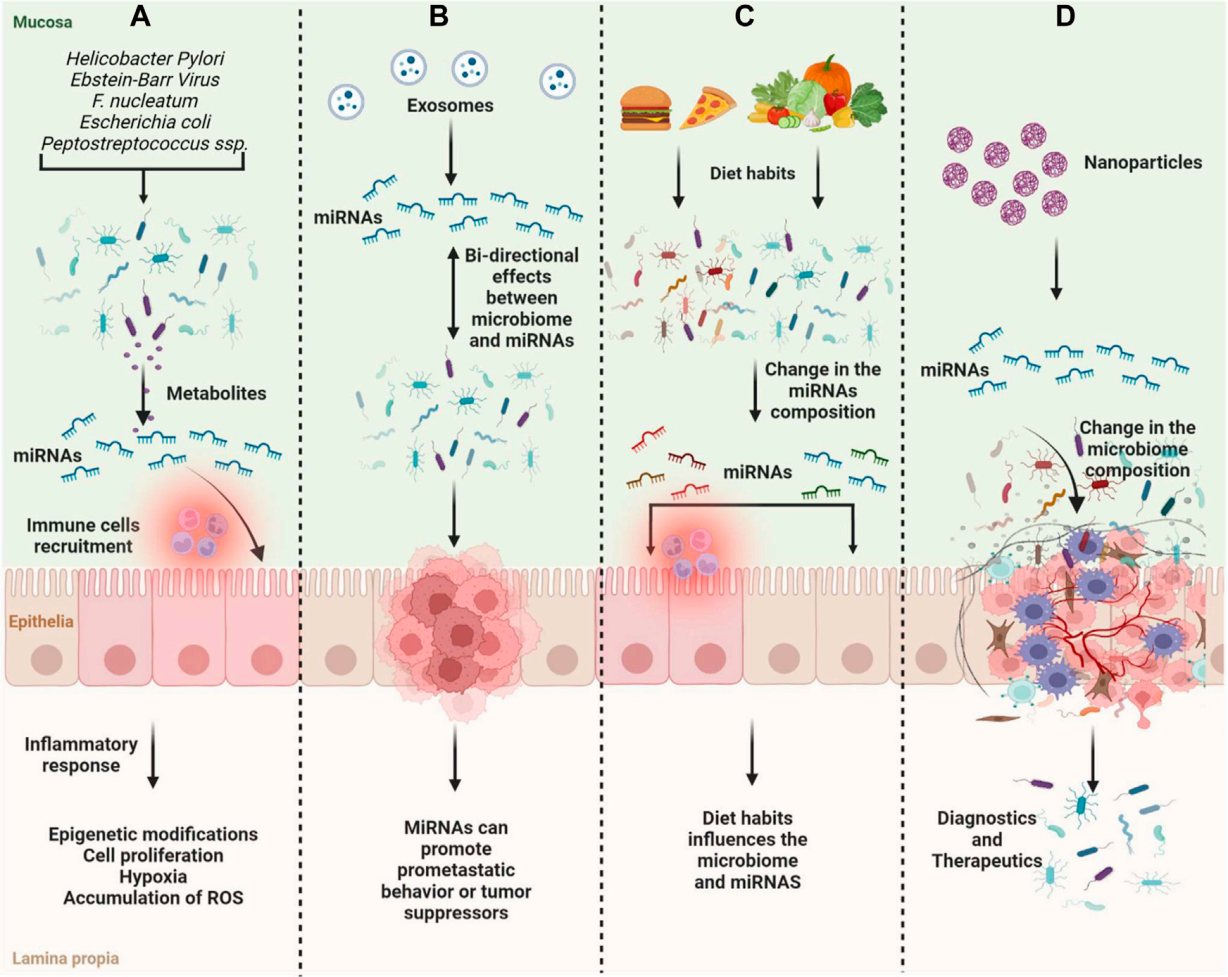
Scheme of the gut microbiome and miRNAs on tumor suppression and oncogenesis. **(A)** Microbiome compositions can induce changes in miRNA expression and induce inflammation, immune recruitment, and epigenetic modifications associated with cancer progression; **(B)** MiRNAs and microbiota have bi-directional effects on cancer development; **(C)** Diet habits can influence the microbiome composition along with miRNA expression; **(D)** ncRNAs can be used as therapeutics influencing the microbiome composition or as diagnostics.

Like *Fusobacterium nucleatum*, it has been shown that different *Escherichia coli* bacteria contribute favorably to CRC carcinogenesis in a way that is reliant on miRNA (Cougnoux et al., 2014). Tan et al. demonstrated that probiotics could inhibit the expression of the oncogenes hsa-mir-153 and hsa-mir-429 and increase the expression of the tumor suppressors hsa-mir-140 and hsa-mir-132, whereas pathobionts could increase the expression of the oncogenes hsa-mir-223, hsa-mir-96, and hsa-mir-106a (Tan et al., 2021). The research revealed that the development of colorectal cancer and microbiota-mediated colorectal carcinogenesis depend on these miRNAs (Figure 2A, B).

LncRNA is a subclass of non-coding RNA that has been linked to intestinal microbiota, tumor progression, and colorectal carcinogenesis (Hayashi et al., 2003; Delgado et al., 2006). Four lncRNAs, including LINC00355, KCNQ1OT1, LINC00491, and HOTAIR, were found in the probiotic and pathobiont environments while two lncRNAs, including LINC00355 and KCNQ1OT1, were found in the pathogenic environment, according to Tan et al. Patients with CRC who express these lncRNAs strongly have poor prognostic value (Tsai et al., 2020).

Diet and dietary habits influence the microbiome, which is important in triggering the association between cancer and diet (Guz et al., 2021). On the other hand, tumor-associated-miRNAs can regulate the growth and composition of the gut microbiota along with the change in the metabolism (Yuan et al., 2019). Metabolites produced by the microbiome can change gene expression including miRNAs (Figure 2C).

The miRNA expression is regulated by gut microbiota, and aberrations in their expression can lead to pathological processes involved in cancer development and progression. MiR-17-92, miR-21, and miR-503 result to be more expressed in tumoral tissues compared with normal (Yuan et al., 2018). MiR-21 reduces the expression of RAS p21 and programmed cell death-4. In CRC, infection of *Fusobacterium nucleatum* increases the expression of miR-21 and down-modulation of miR-18a and miR-4802 (Wang et al., 2021). Crosstalk between microRNA-microbiota in the gut plays a pivotal role in gut homeostasis. microRNAs secreted by intestinal epithelial cells or diet-derived can influence the microbiota composition. There is a bi-directional regulation of miRNAs released and microbiota composition (Bi et al., 2020). Delivery of miRNAs may be explored as a therapeutic strategy due to the potential therapeutic interaction between miRNAs and bacteria (Figure 2D). Despite many studies, the gut microbiome and ncRNAs’ relationship and pathway mechanisms are still not fully known. Clarifying this process is very important for understanding the biology of cancer and developing treatment options.

## 10 Conclusion

It has been shown that changes in the microbiota that cause dysbiosis are associated with various diseases, especially cancer. In recent years, it has been suggested that the gut microbiota, especially dysbiosis, or some specific microorganism species play an important role in the development of cancer, especially GI cancers. In this context, it is not easy to come to a definitive general judgment. Because many factors modulate this relationship, both host and microorganism, as well as the environment.

The research has made important progress in the study of microbiota to facilitate the diagnosis and treatment of human diseases and to prevent them. Targeted microbiota interventions with natural foods and nutraceuticals including biotics may be used to prevent cancer, particularly in patients at risk and specific manipulations of the microbiota could be introduced into the clinic as an adjuvant regimen to increase efficacy and ideally to reduce the side effects of anti-cancer treatments. Of course, the most important issue in this application is to do this in expert monitoring and to consider the possible profit/loss ratio.

The accumulation of scientific evidence offers opportunities for the development of new applications in cancer diagnosis and management. The microorganisms involved in the pathogenesis can offer valid biomarkers to get more information about the disease, the response to treatment, and its handling. Recent studies report that ncRNAs play an important role in cancer development by regulating several mechanisms involved in cell proliferation, differentiation, and apoptosis, including gut microbiome composition. ncRNAs represent promising approaches as diagnostics and therapeutics to explore in the future. However, further investigations are needed to deeply understand the role of ncRNAs in GI cancer.

While waiting for further evidence to introduce the microbiota among the tools of precision medicine and nutrition, the most immediate approach is to dietary intervention enriching with fibers, and foods rich in bioactive substances and through pro-prebiotic foods, *etc.*, limiting the consumption of processed foods and some certain type of animal foods. Along the same line, there is important evidence that the MedDiet is has a protective effect on GI cancers by modulating the microbiota. In addition to healthy nutrition practices, rational drug use strategies should be developed, and especially antibiotic treatments should be applied rationally to prevent all health problems related to dysbiosis, including GI cancers. Considering all these issues, it will be more realistic to develop microbiota-oriented strategies, especially in the prevention and treatment of GI cancers.

## Glossary

NCDs: Non-communicable diseases
GI: Gastrointestinal
DNA: Deoxyribonucleic acid
nc-RNAs: Non-coding RNAs
16S rRNA: 16S ribosomal RNA
SCFAs: Short-chain fatty acids
IBD: Inflammatory bowel disease
Tregs: Regulatory T cells
NK: Natural killer
HIF: Hypoxia-induced factor
LPS: Lipopolysaccharide metabolism
TAAs: Tumor-associated antigens
TNF: Tumor Necrosis Factor
IFN-γ: Interferon-gamma
MedDiet: Mediterranean diet
MUFAs: Monounsaturated fatty acids
PUFAs: Polyunsaturated fatty acids
EPIC: European Prospective Investigation into Cancer and Nutrition
DCA: Deoxycholic acid
ITCs: Isothiocyanates
COX-2: Cyclooxygenase-2
AP-1: Activator protein-1
iNOS: Inducible nitric oxide synthase
ISAPP: International Scientific Association of Probiotics and Prebiotics
PD-1: Anti-programmed death 1
CTLA4: Cytotoxic T-lymphocyte–associated antigen 4
TLRs: Toll-Like Receptors
PAMPs: Pathogen-associated molecular patterns
HDAC: Histone deacetylases
ROS: Reactive oxygen specie
SFN: Sulforaphane
GIT: Gastrointestinal tract
ncRNA: non-coding RNA
NT: Nucleotides

## References

[B1] Abreu M. T. Peek R. M. Jr (2014). Gastrointestinal malignancy and the microbiome. Gastroenterology 146 (6), 1534–1546. e3. 10.1053/j.gastro.2014.01.001 24406471 PMC3995897

[B2] Agans R. Rigsbee L. Kenche H. Michail S. Khamis H. J. Paliy O. (2011). Distal gut microbiota of adolescent children is different from that of adults. FEMS Microbiol. Ecol. 77 (2), 404–412. 10.1111/j.1574-6941.2011.01120.x 21539582 PMC4502954

[B3] Allali I. Delgado S. Marron P. I. Astudillo A. Yeh J. J. Ghazal H. (2015). Gut microbiome compositional and functional differences between tumor and non-tumor adjacent tissues from cohorts from the US and Spain. Gut microbes 6 (3), 161–172. 10.1080/19490976.2015.1039223 25875428 PMC4615176

[B4] Anand P. Kunnumakara A. B. Sundaram C. Harikumar K. B. Tharakan S. T. Lai O. S. (2008). Cancer is a preventable disease that requires major lifestyle changes. Pharm. Res. 25 (9), 2097–2116. 10.1007/s11095-008-9661-9 18626751 PMC2515569

[B5] Arpaia N. Campbell C. Fan X. Dikiy S. van der Veeken J. deRoos P. (2013). Metabolites produced by commensal bacteria promote peripheral regulatory T-cell generation. Nature 504 (7480), 451–455. 10.1038/nature12726 24226773 PMC3869884

[B6] Arthur J. C. Perez-Chanona E. Muhlbauer M. Tomkovich S. Uronis J. M. Fan T. J. (2012). Intestinal inflammation targets cancer-inducing activity of the microbiota. Science 338 (6103), 120–123. 10.1126/science.1224820 22903521 PMC3645302

[B7] Arumugam M. Raes J. Pelletier E. Le Paslier D. Yamada T. Mende D. R. (2011). Enterotypes of the human gut microbiome. Nature 473 (7346), 174–180. 10.1038/nature09944 21508958 PMC3728647

[B8] Atarashi K. Suda W. Luo C. Kawaguchi T. Motoo I. Narushima S. (2017). Ectopic colonization of oral bacteria in the intestine drives TH1 cell induction and inflammation. Science 358 (6361), 359–365. 10.1126/science.aan4526 29051379 PMC5682622

[B9] Atarashi K. Tanoue T. Shima T. Imaoka A. Kuwahara T. Momose Y. (2011). Induction of colonic regulatory T cells by indigenous Clostridium species. Science 331 (6015), 337–341. 10.1126/science.1198469 21205640 PMC3969237

[B10] Aviles-Jimenez F. Vazquez-Jimenez F. Medrano-Guzman R. Mantilla A. Torres J. (2014). Stomach microbiota composition varies between patients with non-atrophic gastritis and patients with intestinal type of gastric cancer. Sci. Rep. 4, 4202. 10.1038/srep04202 24569566 PMC3935187

[B11] Backhed F. Ley R. E. Sonnenburg J. L. Peterson D. A. Gordon J. I. (2005). Host-bacterial mutualism in the human intestine. Science 307 (5717), 1915–1920. 10.1126/science.1104816 15790844

[B12] Baxter N. T. Mtt R. Rogers M. A. Schloss P. D. (2016). Microbiota-based model improves the sensitivity of fecal immunochemical test for detecting colonic lesions. Genome Med. 8 (1), 37. 10.1186/s13073-016-0290-3 27056827 PMC4823848

[B13] Bengmark S. (2001). Pre-pro- and synbiotics. Curr. Opin. Clin. Nutr. metabolic care 4 (6), 571–579. 10.1097/00075197-200111000-00019 11706296

[B14] Berggren A. M. Nyman E. M. Lundquist I. Bjorck I. M. (1996). Influence of orally and rectally administered propionate on cholesterol and glucose metabolism in obese rats. Br. J. Nutr. 76 (2), 287–294. 10.1079/bjn19960032 8813902

[B15] Bernet M. F. Brassart D. Neeser J. R. Servin A. L. (1994). Lactobacillus acidophilus LA 1 binds to cultured human intestinal cell lines and inhibits cell attachment and cell invasion by enterovirulent bacteria. Gut 35 (4), 483–489. 10.1136/gut.35.4.483 8174985 PMC1374796

[B16] Beslenme B. D. Z. Besinler F. (2017). Mikrobiyota. Tüba-mikrobiyota ve i?nsan sağlığı sempozyumu raporu. Ankara, Turkey: Türkiye Bilimler Akademisi.

[B17] Bi K. Zhang X. Chen W. Diao H. (2020). MicroRNAs regulate intestinal immunity and gut microbiota for gastrointestinal health: A comprehensive review. Genes (Basel) 11 (9), 1075. 10.3390/genes11091075 32932716 PMC7564790

[B18] Binda S. Hill C. Johansen E. Obis D. Pot B. Sanders M. E. (2020). Criteria to qualify microorganisms as “probiotic” in foods and dietary supplements. Front. Microbiol. 11 (1662), 1662. 10.3389/fmicb.2020.01662 32793153 PMC7394020

[B19] Bode L. M. Bunzel D. Huch M. Cho G. S. Ruhland D. Bunzel M. (2013). *In vivo* and *in vitro* metabolism of trans-resveratrol by human gut microbiota. Am. J. Clin. Nutr. 97 (2), 295–309. 10.3945/ajcn.112.049379 23283496

[B20] Borges-Canha M. Portela-Cidade J. P. Dinis-Ribeiro M. Leite-Moreira A. F. Pimentel-Nunes P. (2015). Role of colonic microbiota in colorectal carcinogenesis: A systematic review. Rev. espanola enfermedades Dig. organo Of. Soc. Espanola Patol. Dig. 107 (11), 659–671. 10.17235/reed.2015.3830/2015 26541655

[B21] Boursi B. Mamtani R. Haynes K. Yang Y. X. (2015). Recurrent antibiotic exposure may promote cancer formation-Another step in understanding the role of the human microbiota? Eur. J. cancer 51 (17), 2655–2664. 10.1016/j.ejca.2015.08.015 26338196 PMC4663115

[B22] Brawner K. M. Morrow C. D. Smith P. D. (2014). Gastric microbiome and gastric cancer. Cancer J. 20 (3), 211–216. 10.1097/PPO.0000000000000043 24855010 PMC4149312

[B23] Brinkworth G. D. Noakes M. Clifton P. M. Bird A. R. (2009). Comparative effects of very low-carbohydrate, high-fat and high-carbohydrate, low-fat weight-loss diets on bowel habit and faecal short-chain fatty acids and bacterial populations. Br. J. Nutr. 101 (10), 1493–1502. 10.1017/S0007114508094658 19224658

[B24] Brook I. (1999). Bacterial interference. Crit. Rev. Microbiol. 25 (3), 155–172. 10.1080/10408419991299211 10524328

[B25] Bultman S. J. (2014). Emerging roles of the microbiome in cancer. Carcinogenesis 35 (2), 249–255. 10.1093/carcin/bgt392 24302613 PMC3908754

[B26] Bultman S. J. (2016). The microbiome and its potential as a cancer preventive intervention. Seminars Oncol. 43, 97–106. 10.1053/j.seminoncol.2015.09.001 PMC4789109 26970128

[B27] Castellarin M. Warren R. L. Freeman J. D. Dreolini L. Krzywinski M. Strauss J. (2012). Fusobacterium nucleatum infection is prevalent in human colorectal carcinoma. Genome Res. 22 (2), 299–306. 10.1101/gr.126516.111 22009989 PMC3266037

[B28] Chang H. Kim N. Park J. H. Nam R. H. Choi Y. J. Lee H. S. (2015). Different microRNA expression levels in gastric cancer depending on *Helicobacter pylori* infection. Gut Liver 9 (2), 188–196. 10.5009/gnl13371 25167801 PMC4351025

[B29] Chang P. V. Hao L. Offermanns S. Medzhitov R. (2014). The microbial metabolite butyrate regulates intestinal macrophage function via histone deacetylase inhibition. Proc. Natl. Acad. Sci. U. S. A. 111 (6), 2247–2252. 10.1073/pnas.1322269111 24390544 PMC3926023

[B30] Chen W. Liu F. Ling Z. Tong X. Xiang C. (2012). Human intestinal lumen and mucosa-associated microbiota in patients with colorectal cancer. PloS one 7 (6), e39743. 10.1371/journal.pone.0039743 22761885 PMC3386193

[B31] Chen W. J. Anderson J. W. Jennings D. (1984). Propionate may mediate the hypocholesterolemic effects of certain soluble plant fibers in cholesterol-fed rats. Proc. Soc. Exp. Biol. Med. Soc. Exp. Biol. Med. 175 (2), 215–218. 10.3181/00379727-175-41791 6320209

[B32] Chen X. Winckler B. Lu M. Cheng H. Yuan Z. Yang Y. (2015). Oral microbiota and risk for esophageal squamous cell carcinoma in a high-risk area of China. PloS one 10 (12), e0143603. 10.1371/journal.pone.0143603 26641451 PMC4671675

[B33] Chong E. S. L. (2014). A potential role of probiotics in colorectal cancer prevention: Review of possible mechanisms of action. World J. Microbiol. Biotechnol. 30 (2), 351–374. 10.1007/s11274-013-1499-6 24068536

[B34] Chung C. S. Chang P. F. Liao C. H. Lee T. H. Chen Y. Lee Y. C. (2016). Differences of microbiota in small bowel and faeces between irritable bowel syndrome patients and healthy subjects. Scand. J. gastroenterology 51 (4), 410–419. 10.3109/00365521.2015.1116107 26595305

[B35] Clarke J. D. Riedl K. Bella D. Schwartz S. J. Stevens J. F. Ho E. (2011). Comparison of isothiocyanate metabolite levels and histone deacetylase activity in human subjects consuming broccoli sprouts or broccoli supplement. J. Agric. food Chem. 59 (20), 10955–10963. 10.1021/jf202887c 21928849 PMC3201700

[B36] Coker O. O. Dai Z. Nie Y. Zhao G. Cao L. Nakatsu G. (2018). Mucosal microbiome dysbiosis in gastric carcinogenesis. Gut 67 (6), 1024–1032. 10.1136/gutjnl-2017-314281 28765474 PMC5969346

[B37] Cotillard A. Kennedy S. P. Kong L. C. Prifti E. Pons N. Le Chatelier E. (2013). Dietary intervention impact on gut microbial gene richness. Nature 500 (7464), 585–588. 10.1038/nature12480 23985875

[B38] Cougnoux A. Dalmasso G. Martinez R. Buc E. Delmas J. Gibold L. (2014). Bacterial genotoxin colibactin promotes colon tumour growth by inducing a senescence-associated secretory phenotype. Gut 63 (12), 1932–1942. 10.1136/gutjnl-2013-305257 24658599

[B39] Cuevas-Ramos G. Petit C. R. Marcq I. Boury M. Oswald E. Nougayrede J. P. (2010). *Escherichia coli* induces DNA damage *in vivo* and triggers genomic instability in mammalian cells. Proc. Natl. Acad. Sci. U. S. A. 107 (25), 11537–11542. 10.1073/pnas.1001261107 20534522 PMC2895108

[B40] Dai Z. Coker O. O. Nakatsu G. Wu W. K. K. Zhao L. Chen Z. (2018). Multi-cohort analysis of colorectal cancer metagenome identified altered bacteria across populations and universal bacterial markers. Microbiome 6 (1), 70. 10.1186/s40168-018-0451-2 29642940 PMC5896039

[B41] David L. A. Maurice C. F. Carmody R. N. Gootenberg D. B. Button J. E. Wolfe B. E. (2014). Diet rapidly and reproducibly alters the human gut microbiome. Nature 505 (7484), 559–563. 10.1038/nature12820 24336217 PMC3957428

[B42] De Filippis F. Pellegrini N. Vannini L. Jeffery I. B. La Storia A. Laghi L. (2016). High-level adherence to a Mediterranean diet beneficially impacts the gut microbiota and associated metabolome. Gut 65 (11), 1812–1821. 10.1136/gutjnl-2015-309957 26416813

[B43] De Filippo C. Cavalieri D. Di Paola M. Ramazzotti M. Poullet J. B. Massart S. (2010). Impact of diet in shaping gut microbiota revealed by a comparative study in children from Europe and rural Africa. Proc. Natl. Acad. Sci. U. S. A. 107 (33), 14691–14696. 10.1073/pnas.1005963107 20679230 PMC2930426

[B44] de Jesus Raposo M. F. de Morais A. M. de Morais R. M. (2016). Emergent sources of prebiotics: Seaweeds and microalgae. Mar. drugs 14 (2), 27. 10.3390/md14020027 26828501 PMC4771980

[B45] De Martel C. Ferlay J. Franceschi S. Vignat J. Bray F. Forman D. (2012). Global burden of cancers attributable to infections in 2008: A review and synthetic analysis. lancet Oncol. 13 (6), 607–615. 10.1016/S1470-2045(12)70137-7 22575588

[B46] DeGruttola A. K. Low D. Mizoguchi A. Mizoguchi E. (2016). Current understanding of dysbiosis in disease in human and animal models. Inflamm. Bowel Dis. 22 (5), 1137–1150. 10.1097/MIB.0000000000000750 27070911 PMC4838534

[B47] Del Chierico F. Vernocchi P. Dallapiccola B. Putignani L. (2014). Mediterranean diet and health: Food effects on gut microbiota and disease control. Int. J. Mol. Sci. 15 (7), 11678–11699. 10.3390/ijms150711678 24987952 PMC4139807

[B48] Delgado S. Suarez A. Mayo B. (2006). Identification of dominant bacteria in feces and colonic mucosa from healthy Spanish adults by culturing and by 16S rDNA sequence analysis. Dig. Dis. Sci. 51 (4), 744–751. 10.1007/s10620-006-3201-4 16614998

[B49] Dethlefsen L. Huse S. Sogin M. L. Relman D. A. (2008). The pervasive effects of an antibiotic on the human gut microbiota, as revealed by deep 16S rRNA sequencing. PLoS Biol. 6 (11), e280. 10.1371/journal.pbio.0060280 19018661 PMC2586385

[B50] Donohoe D. R. Holley D. Collins L. B. Montgomery S. A. Whitmore A. C. Hillhouse A. (2014). A gnotobiotic mouse model demonstrates that dietary fiber protects against colorectal tumorigenesis in a microbiota- and butyrate-dependent manner. Cancer Discov. 4 (12), 1387–1397. 10.1158/2159-8290.CD-14-0501 25266735 PMC4258155

[B51] Drewes J. L. White J. R. Dejea C. M. Fathi P. Iyadorai T. Vadivelu J. (2017). High-resolution bacterial 16S rRNA gene profile meta-analysis and biofilm status reveal common colorectal cancer consortia. NPJ biofilms microbiomes 3, 34. 10.1038/s41522-017-0040-3 29214046 PMC5707393

[B52] Dubin K. Callahan M. K. Ren B. Khanin R. Viale A. Ling L. (2016). Intestinal microbiome analyses identify melanoma patients at risk for checkpoint-blockade-induced colitis. Nat. Commun. 7, 10391. 10.1038/ncomms10391 26837003 PMC4740747

[B53] Eckburg P. B. Bik E. M. Bernstein C. N. Purdom E. Dethlefsen L. Sargent M. (2005). Diversity of the human intestinal microbial flora. Science 308 (5728), 1635–1638. 10.1126/science.1110591 15831718 PMC1395357

[B54] Enck P. Zimmermann K. Rusch K. Schwiertz A. Klosterhalfen S. Frick J. S. (2009). The effects of maturation on the colonic microflora in infancy and childhood. Gastroenterology Res. Pract. 2009, 752401. 10.1155/2009/752401 PMC2744901 19763278

[B55] Eun C. S. Kim B. K. Han D. S. Kim S. Y. Kim K. M. (2014). Differences in gastric mucosal microbiota profiling in patients with chronic gastritis, intestinal metaplasia, and gastric cancer using pyrosequencing methods. Helicobacter 19 (6), 407–416. 10.1111/hel.12145 25052961

[B56] Fallani M. Amarri S. Uusijarvi A. Adam R. Khanna S. Aguilera M. (2011). Determinants of the human infant intestinal microbiota after the introduction of first complementary foods in infant samples from five European centres. Microbiology 157, 1385–1392. 10.1099/mic.0.042143-0 21330436

[B57] Farrell J. J. Zhang L. Zhou H. Chia D. Elashoff D. Akin D. (2012). Variations of oral microbiota are associated with pancreatic diseases including pancreatic cancer. Gut 61 (4), 582–588. 10.1136/gutjnl-2011-300784 21994333 PMC3705763

[B58] Fasanelli F. Giraudo M. T. Vineis P. Fiano V. Fiorito G. Grasso C. (2019). DNA methylation, colon cancer and mediterranean diet: Results from the EPIC-Italy cohort. Epigenetics 14 (10), 977–988. 10.1080/15592294.2019.1629230 31179817 PMC6691992

[B59] Feng Q. Liang S. Jia H. Stadlmayr A. Tang L. Lan Z. (2015). Gut microbiome development along the colorectal adenoma-carcinoma sequence. Nat. Commun. 6, 6528. 10.1038/ncomms7528 25758642

[B60] Flanagan L. Schmid J. Ebert M. Soucek P. Kunicka T. Liska V. (2014). Fusobacterium nucleatum associates with stages of colorectal neoplasia development, colorectal cancer and disease outcome. Eur. J. Clin. Microbiol. Infect. Dis. 33 (8), 1381–1390. 10.1007/s10096-014-2081-3 24599709

[B61] Flemer B. Lynch D. B. Brown J. M. Jeffery I. B. Ryan F. J. Claesson M. J. (2017). Tumour-associated and non-tumour-associated microbiota in colorectal cancer. Gut 66 (4), 633–643. 10.1136/gutjnl-2015-309595 26992426 PMC5529966

[B62] Fong W. Li Q. Yu J. (2020). Gut microbiota modulation: A novel strategy for prevention and treatment of colorectal cancer. Oncogene 39 (26), 4925–4943. 10.1038/s41388-020-1341-1 32514151 PMC7314664

[B63] Frank D. N. St Amand A. L. Feldman R. A. Boedeker E. C. Harpaz N. Pace N. R. (2007). Molecular-phylogenetic characterization of microbial community imbalances in human inflammatory bowel diseases. Proc. Natl. Acad. Sci. U. S. A. 104 (34), 13780–13785. 10.1073/pnas.0706625104 17699621 PMC1959459

[B64] Fuhler G. M. Tyl M. R. Olthof S. G. Lyndsay Drayer A. Blom N. Vellenga E. (2009). Distinct roles of the mTOR components Rictor and Raptor in MO7e megakaryocytic cells. Eur. J. Haematol. 83 (3), 235–245. 10.1111/j.1600-0609.2009.01263.x 19341427

[B65] Fukui H. (2016). Increased intestinal permeability and decreased barrier function: Does it really influence the risk of inflammation? Inflamm. Intest. Dis. 1 (3), 135–145. 10.1159/000447252 29922669 PMC5988153

[B66] Fung K. Y. Cosgrove L. Lockett T. Head R. Topping D. L. (2012). A review of the potential mechanisms for the lowering of colorectal oncogenesis by butyrate. Br. J. Nutr. 108 (5), 820–831. 10.1017/S0007114512001948 22676885

[B67] Furusawa Y. Obata Y. Fukuda S. Endo T. A. Nakato G. Takahashi D. (2013). Commensal microbe-derived butyrate induces the differentiation of colonic regulatory T cells. Nature 504 (7480), 446–450. 10.1038/nature12721 24226770

[B68] Gallo A. Passaro G. Gasbarrini A. Landolfi R. Montalto M. (2016). Modulation of microbiota as treatment for intestinal inflammatory disorders: An uptodate. World J. gastroenterology 22 (32), 7186–7202. 10.3748/wjg.v22.i32.7186 PMC4997632 27621567

[B69] Galluzzi L. Buque A. Kepp O. Zitvogel L. Kroemer G. (2015). Immunological effects of conventional chemotherapy and targeted anticancer agents. Cancer Cell 28 (6), 690–714. 10.1016/j.ccell.2015.10.012 26678337

[B70] Garcia-Mantrana I. Selma-Royo M. Alcantara C. Collado M. C. (2018). Shifts on gut microbiota associated to mediterranean diet adherence and specific dietary intakes on general adult population. Front. Microbiol. 9, 890. 10.3389/fmicb.2018.00890 29867803 PMC5949328

[B71] Giamarellos-Bourboulis E. Tang J. Pyleris E. Pistiki A. Barbatzas C. Brown J. (2015). Molecular assessment of differences in the duodenal microbiome in subjects with irritable bowel syndrome. Scand. J. gastroenterology 50 (9), 1076–1087. 10.3109/00365521.2015.1027261 25865706

[B72] Gibson G. R. Hutkins R. Sanders M. E. Prescott S. L. Reimer R. A. Salminen S. J. (2017). Expert consensus document: The International Scientific Association for Probiotics and Prebiotics (ISAPP) consensus statement on the definition and scope of prebiotics. Nat. Rev. Gastroenterology Hepatology 14 (8), 491–502. 10.1038/nrgastro.2017.75 28611480

[B73] Gibson P. R. Moeller I. Kagelari O. Folino M. Young G. P. (1992). Contrasting effects of butyrate on the expression of phenotypic markers of differentiation in neoplastic and non-neoplastic colonic epithelial cells *in vitro* . J. gastroenterology hepatology 7 (2), 165–172. 10.1111/j.1440-1746.1992.tb00956.x 1571499

[B74] Gibson P. R. Rosella O. Wilson A. J. Mariadason J. M. Rickard K. Byron K. (1999). Colonic epithelial cell activation and the paradoxical effects of butyrate. Carcinogenesis 20 (4), 539–544. 10.1093/carcin/20.4.539 10223179

[B75] Goncalves P. Martel F. (2013). Butyrate and colorectal cancer: The role of butyrate transport. Curr. drug Metab. 14 (9), 994–1008. 10.2174/1389200211314090006 24160296

[B76] Gopalakrishnan V. Spencer C. N. Nezi L. Reuben A. Andrews M. C. Karpinets T. V. (2018). Gut microbiome modulates response to anti-PD1 immunotherapy in melanoma patients. Science 359 (6371), 97–103. 10.1126/science.aan4236 29097493 PMC5827966

[B77] Greig S. L. (2015). Brexpiprazole: First global approval. Drugs 75 (14), 1687–1697. 10.1007/s40265-015-0462-2 26310190

[B78] Groer M. W. Luciano A. A. Dishaw L. J. Ashmeade T. L. Miller E. Gilbert J. A. (2014). Development of the preterm infant gut microbiome: A research priority. Microbiome 2 (1), 38–8. 10.1186/2049-2618-2-38 25332768 PMC4203464

[B79] Gronlund M. M. Lehtonen O. P. Eerola E. Kero P. (1999). Fecal microflora in healthy infants born by different methods of delivery: Permanent changes in intestinal flora after cesarean delivery. J. Pediatr. gastroenterology Nutr. 28 (1), 19–25. 10.1097/00005176-199901000-00007 9890463

[B80] Guarner F. Casellas F. Borruel N. Antolìn M. Videla S. VilasecaMalagelada J. Jr (2003). Gut flora in health and disease. Gut flora health Dis. Lancet 361, 512–519. 10.1016/S0140-6736(03)12489-0 12583961

[B81] Guarner F. Malagelada J. R. (2003). Gut flora in health and disease. Lancet 361 (9356), 512–519. 10.1016/S0140-6736(03)12489-0 12583961

[B82] Guz M. Jeleniewicz W. Malm A. Korona-Glowniak I. (2021). A crosstalk between diet, microbiome and microRNA in epigenetic regulation of colorectal cancer. Nutrients 13 (7), 2428. 10.3390/nu13072428 34371938 PMC8308570

[B83] Harmsen H. J. Wildeboer-Veloo A. C. Raangs G. C. Wagendorp A. A. Klijn N. Bindels J. G. (2000). Analysis of intestinal flora development in breast-fed and formula-fed infants by using molecular identification and detection methods. J. Pediatr. gastroenterology Nutr. 30 (1), 61–67. 10.1097/00005176-200001000-00019 10630441

[B84] Hayashi H. Sakamoto M. Kitahara M. Benno Y. (2003). Molecular analysis of fecal microbiota in elderly individuals using 16S rDNA library and T-RFLP. Microbiol. Immunol. 47 (8), 557–570. 10.1111/j.1348-0421.2003.tb03418.x 14524616

[B85] Hayashi H. Takahashi R. Nishi T. Sakamoto M. Benno Y. (2005). Molecular analysis of jejunal, ileal, caecal and recto-sigmoidal human colonic microbiota using 16S rRNA gene libraries and terminal restriction fragment length polymorphism. J. Med. Microbiol. 54, 1093–1101. 10.1099/jmm.0.45935-0 16192442

[B86] Higdon J. V. Delage B. Williams D. E. Dashwood R. H. (2007). Cruciferous vegetables and human cancer risk: Epidemiologic evidence and mechanistic basis. Pharmacol. Res. 55 (3), 224–236. 10.1016/j.phrs.2007.01.009 17317210 PMC2737735

[B87] Hill C. Guarner F. Reid G. Gibson G. R. Merenstein D. J. Pot B. (2014). Expert consensus document: The International Scientific Association for Probiotics and Prebiotics consensus statement on the scope and appropriate use of the term probiotic. Nat. Rev. Gastroenterology hepatology 11, 506–514. 10.1038/nrgastro.2014.66 24912386

[B88] Hoekstra E. Das A. M. Swets M. Cao W. van der Woude C. J. Bruno M. J. (2016). Increased PTP1B expression and phosphatase activity in colorectal cancer results in a more invasive phenotype and worse patient outcome. Oncotarget 7 (16), 21922–21938. 10.18632/oncotarget.7829 26942883 PMC5008334

[B89] Hold G. L. (2016). Gastrointestinal microbiota and colon cancer. Dig. Dis. 34 (3), 244–250. 10.1159/000443358 27028619

[B90] Hold G. L. Pryde S. E. Russell V. J. Furrie E. Flint H. J. (2002). Assessment of microbial diversity in human colonic samples by 16S rDNA sequence analysis. FEMS Microbiol. Ecol. 39 (1), 33–39. 10.1111/j.1574-6941.2002.tb00904.x 19709182

[B91] Holmes E. Li J. V. Athanasiou T. Ashrafian H. Nicholson J. K. (2011). Understanding the role of gut microbiome-host metabolic signal disruption in health and disease. Trends Microbiol. 19 (7), 349–359. 10.1016/j.tim.2011.05.006 21684749

[B92] Honda K. Littman D. R. (2012). The microbiome in infectious disease and inflammation. Annu. Rev. Immunol. 30, 759–795. 10.1146/annurev-immunol-020711-074937 22224764 PMC4426968

[B93] Hooper L. V. Xu J. Falk P. G. Midtvedt T. Gordon J. I. (1999). A molecular sensor that allows a gut commensal to control its nutrient foundation in a competitive ecosystem. Proc. Natl. Acad. Sci. U. S. A. 96 (17), 9833–9838. 10.1073/pnas.96.17.9833 10449780 PMC22296

[B94] Huycke M. M. Gaskins H. R. (2004). Commensal bacteria, redox stress, and colorectal cancer: Mechanisms and models. Exp. Biol. Med. 229 (7), 586–597. 10.1177/153537020422900702 15229352

[B95] IARC (1994). Schistosomes, liver flukes and *Helicobacter pylori* . IARC Monogr. Eval. Carcinog. Risks Hum. 61, 1–241. 7715068 PMC7681621

[B96] Iida N. Dzutsev A. Stewart C. A. Smith L. Bouladoux N. Weingarten R. A. (2013). Commensal bacteria control cancer response to therapy by modulating the tumor microenvironment. Science 342 (6161), 967–970. 10.1126/science.1240527 24264989 PMC6709532

[B97] Itzkowitz S. H. Harpaz N. (2004). Diagnosis and management of dysplasia in patients with inflammatory bowel diseases. Gastroenterology 126 (6), 1634–1648. 10.1053/j.gastro.2004.03.025 15168373

[B98] Jakszyn P. Bingham S. Pera G. Agudo A. Luben R. Welch A. (2006). Endogenous versus exogenous exposure to N-nitroso compounds and gastric cancer risk in the European Prospective Investigation into Cancer and Nutrition (EPIC-EURGAST) study. Carcinogenesis 27 (7), 1497–1501. 10.1093/carcin/bgl019 16571648

[B99] Jobin C. (2018). Precision medicine using microbiota. Science 359 (6371), 32–34. 10.1126/science.aar2946 29302001

[B100] Jostins L. Ripke S. Weersma R. K. Duerr R. H. McGovern D. P. Hui K. Y. (2012). Host-microbe interactions have shaped the genetic architecture of inflammatory bowel disease. Nature 491 (7422), 119–124. 10.1038/nature11582 23128233 PMC3491803

[B101] Kau A. L. Ahern P. P. Griffin N. W. Goodman A. L. Gordon J. I. (2011). Human nutrition, the gut microbiome and the immune system. Nature 474 (7351), 327–336. 10.1038/nature10213 21677749 PMC3298082

[B102] Khalili H. Håkansson N. Chan S. S. Chen Y. Lochhead P. Ludvigsson J. F. (2020). Adherence to a mediterranean diet is associated with a lower risk of later-onset Crohn's disease: Results from two large prospective cohort studies. Gut 69 (9), 1637–1644. 10.1136/gutjnl-2019-319505 31900290

[B103] Kiselyov A. Bunimovich-Mendrazitsky S. Startsev V. (2015). Treatment of non-muscle invasive bladder cancer with Bacillus Calmette-Guerin (BCG): Biological markers and simulation studies. BBA Clin. 4, 27–34. 10.1016/j.bbacli.2015.06.002 26673853 PMC4661599

[B104] Kleessen B. Hartmann L. Blaut M. (2003). Fructans in the diet cause alterations of intestinal mucosal architecture, released mucins and mucosa-associated bifidobacteria in gnotobiotic rats. Br. J. Nutr. 89 (5), 597–606. 10.1079/BJN2002827 12720580

[B105] Kostic A. D. Chun E. Robertson L. Glickman J. N. Gallini C. A. Michaud M. (2013). Fusobacterium nucleatum potentiates intestinal tumorigenesis and modulates the tumor-immune microenvironment. Cell host microbe 14 (2), 207–215. 10.1016/j.chom.2013.07.007 23954159 PMC3772512

[B106] Kroemer G. Senovilla L. Galluzzi L. Andre F. Zitvogel L. (2015). Natural and therapy-induced immunosurveillance in breast cancer. Nat. Med. 21 (10), 1128–1138. 10.1038/nm.3944 26444637

[B107] Lam S. Y. Yu J. Wong S. H. Peppelenbosch M. P. Fuhler G. M. (2017). The gastrointestinal microbiota and its role in oncogenesis. Best Pract. Res. Clin. gastroenterology 31 (6), 607–618. 10.1016/j.bpg.2017.09.010 29566903

[B108] Larrosa M. Gonzalez-Sarrias A. Garcia-Conesa M. T. Tomas-Barberan F. A. Espin J. C. (2006). Urolithins, ellagic acid-derived metabolites produced by human colonic microflora, exhibit estrogenic and antiestrogenic activities. J. Agric. food Chem. 54 (5), 1611–1620. 10.1021/jf0527403 16506809

[B109] Le D. T. Wang-Gillam A. Picozzi V. Greten T. F. Crocenzi T. Springett G. (2015). Safety and survival with GVAX pancreas prime and Listeria Monocytogenes-expressing mesothelin (CRS-207) boost vaccines for metastatic pancreatic cancer. J. Clin. Oncol. official J. Am. Soc. Clin. Oncol. 33 (12), 1325–1333. 10.1200/JCO.2014.57.4244 PMC4397277 25584002

[B110] Lertpiriyapong K. Whary M. T. Muthupalani S. Lofgren J. L. Gamazon E. R. Feng Y. (2014). Gastric colonisation with a restricted commensal microbiota replicates the promotion of neoplastic lesions by diverse intestinal microbiota in the *Helicobacter pylori* INS-GAS mouse model of gastric carcinogenesis. Gut 63 (1), 54–63. 10.1136/gutjnl-2013-305178 23812323 PMC4023484

[B111] Ley R. E. Backhed F. Turnbaugh P. Lozupone C. A. Knight R. D. Gordon J. I. (2005). Obesity alters gut microbial ecology. Proc. Natl. Acad. Sci. U. S. A. 102 (31), 11070–11075. 10.1073/pnas.0504978102 16033867 PMC1176910

[B112] Liang Q. Chiu J. Chen Y. Huang Y. Higashimori A. Fang J. (2017). Fecal bacteria act as novel biomarkers for noninvasive diagnosis of colorectal cancer. Clin. cancer Res. official J. Am. Assoc. Cancer Res. 23 (8), 2061–2070. 10.1158/1078-0432.CCR-16-1599 27697996

[B113] Lievin V. Peiffer I. Hudault S. Rochat F. Brassart D. Neeser J. R. (2000). Bifidobacterium strains from resident infant human gastrointestinal microflora exert antimicrobial activity. Gut 47 (5), 646–652. 10.1136/gut.47.5.646 11034580 PMC1728100

[B114] Louis P. Hold G. L. Flint H. J. (2014). The gut microbiota, bacterial metabolites and colorectal cancer. Nat. Rev. Microbiol. 12 (10), 661–672. 10.1038/nrmicro3344 25198138

[B115] Lu Y. Chen J. Zheng J. Hu G. Wang J. Huang C. (2016). Mucosal adherent bacterial dysbiosis in patients with colorectal adenomas. Sci. Rep. 6, 26337. 10.1038/srep26337 27194068 PMC4872055

[B116] Lundberg J. O. Weitzberg E. Lundberg J. M. Alving K. (1994). Intragastric nitric oxide production in humans: Measurements in expelled air. Gut 35 (11), 1543–1546. 10.1136/gut.35.11.1543 7828969 PMC1375608

[B117] Lynch S. V. Pedersen O. (2016). The human intestinal microbiome in health and disease. N. Engl. J. Med. 375 (24), 2369–2379. 10.1056/NEJMra1600266 27974040

[B118] Macfarlane G. T. Cummings J. H. Allison C. (1986). Protein degradation by human intestinal bacteria. J. general Microbiol. 132 (6), 1647–1656. 10.1099/00221287-132-6-1647 3543210

[B119] Marchesi J. R. Dutilh B. E. Hall N. Peters W. H. Roelofs R. Boleij A. (2011). Towards the human colorectal cancer microbiome. PloS one 6 (5), e20447. 10.1371/journal.pone.0020447 21647227 PMC3101260

[B120] Mayer Rj P. C. E. (2003). Esophageal cancer. Esophageal cancer NEngl JMed 349, 2241–2252. 10.1056/NEJMra035010 14657432

[B121] Mendis M. Leclerc E. Simsek S. (2016). Arabinoxylans, gut microbiota and immunity. Carbohydr. Polym. 139, 159–166. 10.1016/j.carbpol.2015.11.068 26794959

[B122] Miękus N. Marszałek K. Podlacha M. Iqbal A. Puchalski C. Świergiel A. H. (2020). Health benefits of plant-derived sulfur compounds, glucosinolates, and organosulfur compounds. Molecules 25 (17), 3804. 10.3390/molecules25173804 32825600 PMC7503525

[B123] Mima K. Nishihara R. Qian Z. R. Cao Y. Sukawa Y. Nowak J. A. (2016). Fusobacterium nucleatum in colorectal carcinoma tissue and patient prognosis. Gut 65 (12), 1973–1980. 10.1136/gutjnl-2015-310101 26311717 PMC4769120

[B124] Mima K. Sukawa Y. Nishihara R. Qian Z. R. Yamauchi M. Inamura K. (2015). Fusobacterium nucleatum and T cells in colorectal carcinoma. JAMA Oncol. 1 (5), 653–661. 10.1001/jamaoncol.2015.1377 26181352 PMC4537376

[B125] Nakatsu G. Li X. Zhou H. Sheng J. Wong S. H. Wu W. K. (2015). Gut mucosal microbiome across stages of colorectal carcinogenesis. Nat. Commun. 6, 8727. 10.1038/ncomms9727 26515465 PMC4640069

[B126] Naylor G. Axon A. (2003). Role of bacterial overgrowth in the stomach as an additional risk factor for gastritis. Can. J. gastroenterology = J. Can. de gastroenterologie 17, 13B–17B. 10.1155/2003/350347 12845343

[B127] O'sullivan M. Thornton G. O'sullivan G. Collins J. (1992). Probiotic bacteria: Myth or reality? Trends food Sci. Technol. 3, 309–314. 10.1016/s0924-2244(10)80018-4

[B128] Oikonomopoulou K. Brinc D. Kyriacou K. Diamandis E. P. (2013). Infection and cancer: Revaluation of the hygiene hypothesis. Clin. cancer Res. official J. Am. Assoc. Cancer Res. 19 (11), 2834–2841. 10.1158/1078-0432.CCR-12-3661 23536438

[B129] O’Keefe S. Li J. Lahti L. Ou J. Carbonero F. Mohammed K. (2015). Fat, fibre and cancer risk in African Americans and rural Africans. Nat. Commun. 6, 6342–6355. 10.1038/ncomms7342 25919227 PMC4415091

[B130] Ou J. Carbonero F. Zoetendal E. G. DeLany J. P. Wang M. Newton K. (2013). Diet, microbiota, and microbial metabolites in colon cancer risk in rural Africans and African Americans. Am. J. Clin. Nutr. 98 (1), 111–120. 10.3945/ajcn.112.056689 23719549 PMC3683814

[B131] Palmer C. Bik E. M. DiGiulio D. B. Relman D. A. Brown P. O. (2007). Development of the human infant intestinal microbiota. PLoS Biol. 5 (7), e177. 10.1371/journal.pbio.0050177 17594176 PMC1896187

[B132] Parchem K. Piekarska A. Bartoszek A. (2020). “Chapter 3 - enzymatic activities behind degradation of glucosinolates,” in Glucosinolates: Properties, recovery, and applications. Editor Galanakis C. M. (Cambridge: Academic Press).

[B133] Peek R. M. Jr. Blaser M. J. (2002). *Helicobacter pylori* and gastrointestinal tract adenocarcinomas. Nat. Rev. Cancer 2 (1), 28–37. 10.1038/nrc703 11902583

[B134] Peek R. M. Jr. Crabtree J. E. (2006). Helicobacter infection and gastric neoplasia. J. pathology 208 (2), 233–248. 10.1002/path.1868 16362989

[B135] Peters B. A. Wu J. Pei Z. Yang L. Purdue M. P. Freedman N. D. (2017). Oral microbiome composition reflects prospective risk for esophageal cancers. Cancer Res. 77 (23), 6777–6787. 10.1158/0008-5472.CAN-17-1296 29196415 PMC5726431

[B136] Pflug N. Kluth S. Vehreschild J. J. Bahlo J. Tacke D. Biehl L. (2016). Efficacy of antineoplastic treatment is associated with the use of antibiotics that modulate intestinal microbiota. Oncoimmunology 5 (6), e1150399. 10.1080/2162402X.2016.1150399 27471619 PMC4938364

[B137] Pickard J. M. Zeng M. Y. Caruso R. Núñez G. (2017). Gut microbiota: Role in pathogen colonization, immune responses, and inflammatory disease. Immunol. Rev. 279 (1), 70–89. 10.1111/imr.12567 28856738 PMC5657496

[B138] Pierro A. van Saene H. K. Donnell S. C. Hughes J. Ewan C. Nunn A. J. (1996). Microbial translocation in neonates and infants receiving long-term parenteral nutrition. Archives Surg. 131 (2), 176–179. 10.1001/archsurg.1996.01430140066018 8611075

[B139] Ploger S. Stumpff F. Penner G. B. Schulzke J. D. Gabel G. Martens H. (2012). Microbial butyrate and its role for barrier function in the gastrointestinal tract. Ann. N. Y. Acad. Sci. 1258, 52–59. 10.1111/j.1749-6632.2012.06553.x 22731715

[B140] Plummer M. de Martel C. Vignat J. Ferlay J. Bray F. Franceschi S. (2016). Global burden of cancers attributable to infections in 2012: A synthetic analysis. Lancet Glob. health 4 (9), e609–e616. 10.1016/S2214-109X(16)30143-7 27470177

[B141] Polk D. B. Peek R. M. Jr. (2010). *Helicobacter pylori*: Gastric cancer and beyond. Nat. Rev. Cancer 10 (6), 403–414. 10.1038/nrc2857 20495574 PMC2957472

[B142] Qin J. Li R. Raes J. Arumugam M. Burgdorf K. S. Manichanh C. (2010). A human gut microbial gene catalogue established by metagenomic sequencing. Nature 464 (7285), 59–65. 10.1038/nature08821 20203603 PMC3779803

[B143] Queiroz K. C. Milani R. Ruela-de-Sousa R. R. Fuhler G. M. Justo G. Z. Zambuzzi W. F. (2012). Violacein induces death of resistant leukaemia cells via kinome reprogramming, endoplasmic reticulum stress and Golgi apparatus collapse. PloS one 7 (10), e45362. 10.1371/journal.pone.0045362 23071514 PMC3469566

[B144] Rabah H. Rosa do Carmo F. L. Jan G. (2017). Dairy propionibacteria: Versatile probiotics. Microorganisms 5 (2), 24. 10.3390/microorganisms5020024 28505101 PMC5488095

[B145] Rad A. H. Aghebati-Maleki L. Kafil H. S. Abbasi A. (2021). Molecular mechanisms of postbiotics in colorectal cancer prevention and treatment. Crit. Rev. food Sci. Nutr. 61 (11), 1787–1803. 10.1080/10408398.2020.1765310 32410512

[B146] Raoult D. (2008). Obesity pandemics and the modification of digestive bacterial flora. Eur. J. Clin. Microbiol. Infect. Dis. official Publ. Eur. Soc. Clin. Microbiol. 27 (8), 631–634. 10.1007/s10096-008-0490-x 18322715

[B147] Rastall R. A. Gibson G. R. (2015). Recent developments in prebiotics to selectively impact beneficial microbes and promote intestinal health. Curr. Opin. Biotechnol. 32, 42–46. 10.1016/j.copbio.2014.11.002 25448231

[B148] Rooney M. S. Shukla S. A. Wu C. J. Getz G. Hacohen N. (2015). Molecular and genetic properties of tumors associated with local immune cytolytic activity. Cell 160 (1-2), 48–61. 10.1016/j.cell.2014.12.033 25594174 PMC4856474

[B149] Routy B. Le Chatelier E. Derosa L. Duong C. P. M. Alou M. T. Daillere R. (2018). Gut microbiome influences efficacy of PD-1-based immunotherapy against epithelial tumors. Science 359 (6371), 91–97. 10.1126/science.aan3706 29097494

[B150] Rubinstein M. R. Wang X. Liu W. Hao Y. Cai G. Han Y. W. (2013). Fusobacterium nucleatum promotes colorectal carcinogenesis by modulating E-cadherin/β-catenin signaling via its FadA adhesin. Cell host microbe 14 (2), 195–206. 10.1016/j.chom.2013.07.012 23954158 PMC3770529

[B151] Russell W. R. Gratz S. W. Duncan S. H. Holtrop G. Ince J. Scobbie L. (2011). High-protein, reduced-carbohydrate weight-loss diets promote metabolite profiles likely to be detrimental to colonic health. Am. J. Clin. Nutr. 93 (5), 1062–1072. 10.3945/ajcn.110.002188 21389180

[B152] Salminen S. Collado M. C. Endo A. Hill C. Lebeer S. Quigley E. M. M. (2021). The International Scientific Association of Probiotics and Prebiotics (ISAPP) consensus statement on the definition and scope of postbiotics. Nat. Rev. Gastroenterology hepatology 18 (9), 649–667. 10.1038/s41575-021-00440-6 33948025 PMC8387231

[B153] Savage D. C. (1977). Microbial ecology of the gastrointestinal tract. Annu. Rev. Microbiol. 31, 107–133. 10.1146/annurev.mi.31.100177.000543 334036

[B154] Schulpen M. van den Brandt P. A. (2020). Mediterranean diet adherence and risk of colorectal cancer: The prospective Netherlands cohort study. Eur. J. Epidemiol. 35 (1), 25–35. 10.1007/s10654-019-00549-8 31494792 PMC7058569

[B155] Scott K. P. Gratz S. W. Sheridan P. O. Flint H. J. Duncan S. H. (2013). The influence of diet on the gut microbiota. Pharmacol. Res. 69 (1), 52–60. 10.1016/j.phrs.2012.10.020 23147033

[B156] Sears C. L. Pardoll D. M. (2011). Perspective: Alpha-bugs, their microbial partners, and the link to colon cancer. J. Infect. Dis. 203 (3), 306–311. 10.1093/jinfdis/jiq061 21208921 PMC3071114

[B157] Sekirov I. Russell S. L. Antunes L. C. Finlay B. B. (2010). Gut microbiota in health and disease. Physiol. Rev. 90 (3), 859–904. 10.1152/physrev.00045.2009 20664075

[B158] Shaw S. Y. Blanchard J. F. Bernstein C. N. (2010). Association between the use of antibiotics in the first year of life and pediatric inflammatory bowel disease. Official J. Am. Coll. Gastroenterology| ACG 105 (12), 2687–2692. 10.1038/ajg.2010.398 20940708

[B159] Shinozaki A. Sakatani T. Ushiku T. Hino R. Isogai M. Ishikawa S. (2010). Downregulation of microRNA-200 in EBV-associated gastric carcinoma. Cancer Res. 70 (11), 4719–4727. 10.1158/0008-5472.CAN-09-4620 20484038

[B160] Siavoshian S. Segain J. P. Kornprobst M. Bonnet C. Cherbut C. Galmiche J. P. (2000). Butyrate and trichostatin A effects on the proliferation/differentiation of human intestinal epithelial cells: Induction of cyclin D3 and p21 expression. Gut 46 (4), 507–514. 10.1136/gut.46.4.507 10716680 PMC1727889

[B161] Singh N. Gurav A. Sivaprakasam S. Brady E. Padia R. Shi H. (2014). Activation of Gpr109a, receptor for niacin and the commensal metabolite butyrate, suppresses colonic inflammation and carcinogenesis. Immunity 40 (1), 128–139. 10.1016/j.immuni.2013.12.007 24412617 PMC4305274

[B162] Sivan A. Corrales L. Hubert N. Williams J. B. Aquino-Michaels K. Earley Z. M. (2015). Commensal Bifidobacterium promotes antitumor immunity and facilitates anti-PD-L1 efficacy. Science 350 (6264), 1084–1089. 10.1126/science.aac4255 26541606 PMC4873287

[B163] Smith P. M. Howitt M. R. Panikov N. Michaud M. Gallini C. A. Bohlooly-y M. (2013). The microbial metabolites, short-chain fatty acids, regulate colonic Treg cell homeostasis. Science 341 (6145), 569–573. 10.1126/science.1241165 23828891 PMC3807819

[B164] Sobhani I. Tap J. Roudot-Thoraval F. Roperch J. P. Letulle S. Langella P. (2011). Microbial dysbiosis in colorectal cancer (CRC) patients. PloS one 6 (1), e16393. 10.1371/journal.pone.0016393 21297998 PMC3029306

[B165] Sommer F. Bäckhed F. (2016). Know your neighbor: Microbiota and host epithelial cells interact locally to control intestinal function and physiology. BioEssays 38 (5), 455–464. 10.1002/bies.201500151 26990415

[B166] Sonnenburg E. D. Smits S. A. Tikhonov M. Higginbottom S. K. Wingreen N. S. Sonnenburg J. L. (2016). Diet-induced extinctions in the gut microbiota compound over generations. Nature 529 (7585), 212–215. 10.1038/nature16504 26762459 PMC4850918

[B167] Stoner G. D. Wang L-S. Casto B. C. (2008). Laboratory and clinical studies of cancer chemoprevention by antioxidants in berries. Carcinogenesis 29 (9), 1665–1674. 10.1093/carcin/bgn142 18544560 PMC3246882

[B168] Sun J. Kato I. (2016). Gut microbiota, inflammation and colorectal cancer. Genes and Dis. 3 (2), 130–143. 10.1016/j.gendis.2016.03.004 PMC5221561 28078319

[B169] Sun K. Jia K. Lv H. Wang S. Q. Wu Y. Lei H. (2020). EBV-Positive gastric cancer: Current knowledge and future perspectives. Front. Oncol. 10, 583463. 10.3389/fonc.2020.583463 33381453 PMC7769310

[B170] Takiishi T. Fenero C. I. M. Câmara N. O. S. (2017). Intestinal barrier and gut microbiota: Shaping our immune responses throughout life. Tissue Barriers 5 (4), e1373208–e. 10.1080/21688370.2017.1373208 28956703 PMC5788425

[B171] Tan X. Mao L. Huang C. Yang W. Guo J. Chen Z. (2021). Comprehensive analysis of lncRNA-miRNA-mRNA regulatory networks for microbiota-mediated colorectal cancer associated with immune cell infiltration. Bioengineered 1, 3410–3425. 10.1080/21655979.2021.1940614 PMC8806860 34227920

[B172] Taur Y. Pamer E. G. (2016). Microbiome mediation of infections in the cancer setting. Genome Med. 8 (1), 40. 10.1186/s13073-016-0306-z 27090860 PMC4835935

[B173] Tian S. Liu X. Lei P. Zhang X. Shan Y. (2018). Microbiota: A mediator to transform glucosinolate precursors in cruciferous vegetables to the active isothiocyanates. J. Sci. food Agric. 98 (4), 1255–1260. 10.1002/jsfa.8654 28869285

[B174] Tilg H. Adolph T. E. Gerner R. R. Moschen A. R. (2018). The intestinal microbiota in colorectal cancer. Cancer Cell 33 (6), 954–964. 10.1016/j.ccell.2018.03.004 29657127

[B175] Tjalsma H. Boleij A. Marchesi J. R. Dutilh B. E. (2012). A bacterial driver-passenger model for colorectal cancer: Beyond the usual suspects. Nat. Rev. Microbiol. 10 (8), 575–582. 10.1038/nrmicro2819 22728587

[B176] Tomasello G. Mazzola M. Leone A. Sinagra E. Zummo G. Farina F. (2016). Nutrition, oxidative stress and intestinal dysbiosis: Influence of diet on gut microbiota in inflammatory bowel diseases. Biomed. Pap. Med. Fac. Univ. Palacky, Olomouc, Czechoslov. 160 (4), 461–466. 10.5507/bp.2016.052 27812084

[B177] Torres P. J. Fletcher E. M. Gibbons S. M. Bouvet M. Doran K. S. Kelley S. T. (2015). Characterization of the salivary microbiome in patients with pancreatic cancer. PeerJ 3, e1373. 10.7717/peerj.1373 26587342 PMC4647550

[B178] Tözün A. (2019). Bağırsak mikrobiyatası gastrointestinal kanserler. İSTANBUL: Acibadem Mehmet Ali Aydinlar University Research Information System.

[B179] Tsai C. C. Chen T. Y. Tsai K. J. Lin M. W. Hsu C. Y. Wu D. C. (2020). NF-κB/miR-18a-3p and miR-4286/BZRAP1 axis may mediate carcinogenesis in Helicobacter pylori-associated gastric cancer. Biomed. Pharmacother. 132, 110869. 10.1016/j.biopha.2020.110869 33113427

[B180] Tsigalou C. Paraschaki A. Karvelas A. Kantartzi K. Gagali K. Tsairidis D. (2021). Gut microbiome and Mediterranean diet in the context of obesity. Current knowledge, perspectives and potential therapeutic targets. Metab. Open 9, 100081. 10.1016/j.metop.2021.100081 PMC7892986 33644741

[B181] Tsoi H. Chu E. S. H. Zhang X. Sheng J. Nakatsu G. Ng S. C. (2017). Peptostreptococcus anaerobius induces intracellular cholesterol biosynthesis in colon cells to induce proliferation and causes dysplasia in mice. Gastroenterology 152 (6), 1419–1433. 10.1053/j.gastro.2017.01.009 28126350

[B182] Turroni F. Foroni E. Pizzetti P. Giubellini V. Ribbera A. Merusi P. (2009). Exploring the diversity of the bifidobacterial population in the human intestinal tract. Appl. Environ. Microbiol. 75 (6), 1534–1545. 10.1128/AEM.02216-08 19168652 PMC2655441

[B183] Vesely M. D. Kershaw M. H. Schreiber R. D. Smyth M. J. (2011). Natural innate and adaptive immunity to cancer. Annu. Rev. Immunol. 29, 235–271. 10.1146/annurev-immunol-031210-101324 21219185

[B184] Vetizou M. Pitt J. M. Daillere R. Lepage P. Waldschmitt N. Flament C. (2015). Anticancer immunotherapy by CTLA-4 blockade relies on the gut microbiota. Science 350 (6264), 1079–1084. 10.1126/science.aad1329 26541610 PMC4721659

[B185] Viaud S. Saccheri F. Mignot G. Yamazaki T. Daillere R. Hannani D. (2013). The intestinal microbiota modulates the anticancer immune effects of cyclophosphamide. Science 342 (6161), 971–976. 10.1126/science.1240537 24264990 PMC4048947

[B186] Vogtmann E. Hua X. Zeller G. Sunagawa S. Voigt A. Y. Hercog R. (2016). Colorectal cancer and the human gut microbiome: Reproducibility with whole-genome shotgun sequencing. PloS one 11 (5), e0155362. 10.1371/journal.pone.0155362 27171425 PMC4865240

[B187] von Martels J. Z. Sadabad M. S. Bourgonje A. R. Blokzijl T. Dijkstra G. Faber K. N. (2017). The role of gut microbiota in health and disease: *In vitro* modeling of host-microbe interactions at the aerobe-anaerobe interphase of the human gut. Anaerobe 44, 3–12. 10.1016/j.anaerobe.2017.01.001 28062270

[B188] Wang F. Yin Q. Chen L. Davis M. M. (2018). Bifidobacterium can mitigate intestinal immunopathology in the context of CTLA-4 blockade. Proc. Natl. Acad. Sci. U. S. A. 115 (1), 157–161. 10.1073/pnas.1712901115 29255057 PMC5776803

[B189] Wang H. B. Wang P. Y. Wang X. Wan Y. L. Liu Y. C. (2012). Butyrate enhances intestinal epithelial barrier function via up-regulation of tight junction protein Claudin-1 transcription. Dig. Dis. Sci. 57 (12), 3126–3135. 10.1007/s10620-012-2259-4 22684624

[B190] Wang M. Ahrne S. Jeppsson B. Molin G. (2005). Comparison of bacterial diversity along the human intestinal tract by direct cloning and sequencing of 16S rRNA genes. FEMS Microbiol. Ecol. 54 (2), 219–231. 10.1016/j.femsec.2005.03.012 16332321

[B191] Wang Q. Ding H. Dong G. Xu L. Jiang F. Mao Q. (2021). Bi-direction effects between microbiome and MiRNAs in carcinogenesis. J. Cancer Res. Clin. Oncol. 147 (5), 1299–1305. 10.1007/s00432-021-03567-w 33765216 PMC11801847

[B192] Wang T. Cai G. Qiu Y. Fei N. Zhang M. Pang X. (2012). Structural segregation of gut microbiota between colorectal cancer patients and healthy volunteers. ISME J. 6 (2), 320–329. 10.1038/ismej.2011.109 21850056 PMC3260502

[B193] Wang X. Heazlewood S. P. Krause D. O. Florin T. H. (2003). Molecular characterization of the microbial species that colonize human ileal and colonic mucosa by using 16S rDNA sequence analysis. J. Appl. Microbiol. 95 (3), 508–520. 10.1046/j.1365-2672.2003.02005.x 12911699

[B194] Wang Z. K. Yang Y. S. (2013). Upper gastrointestinal microbiota and digestive diseases. World J. gastroenterology 19 (10), 1541–1550. 10.3748/wjg.v19.i10.1541 PMC3602471 23539678

[B195] Warnakulasuriya S. (2009). Global epidemiology of oral and oropharyngeal cancer. Oral Oncol. 45 (4-5), 309–316. 10.1016/j.oraloncology.2008.06.002 18804401

[B196] WHO (2020). Handbook on gut microbiome: A global perspective. Turkey: WHO.

[B197] Wollowski I. Rechkemmer G. Pool-Zobel B. L. (2001). Protective role of probiotics and prebiotics in colon cancer. Am. J. Clin. Nutr. 73 (2), 451S–5s. 10.1093/ajcn/73.2.451s 11157356

[B198] Wong J. M. de Souza R. Kendall C. W. Emam A. Jenkins D. J. (2006). Colonic health: Fermentation and short chain fatty acids. J. Clin. gastroenterology 40 (3), 235–243. 10.1097/00004836-200603000-00015 16633129

[B199] Woodmansey E. J. (2007). Intestinal bacteria and ageing. J. Appl. Microbiol. 102 (5), 1178–1186. 10.1111/j.1365-2672.2007.03400.x 17448153

[B200] Wu G. D. Chen J. Hoffmann C. Bittinger K. Chen Y-Y. Keilbaugh S. A. (2011). Linking long-term dietary patterns with gut microbial enterotypes. Science 334 (6052), 105–108. 10.1126/science.1208344 21885731 PMC3368382

[B201] Wu G. D. Compher C. Chen E. Z. Smith S. A. Shah R. D. Bittinger K. (2016). Comparative metabolomics in vegans and omnivores reveal constraints on diet-dependent gut microbiota metabolite production. Gut 65 (1), 63–72. 10.1136/gutjnl-2014-308209 25431456 PMC4583329

[B202] Wu S. Rhee K. J. Albesiano E. Rabizadeh S. Wu X. Yen H. R. (2009). A human colonic commensal promotes colon tumorigenesis via activation of T helper type 17 T cell responses. Nat. Med. 15 (9), 1016–1022. 10.1038/nm.2015 19701202 PMC3034219

[B203] Xu Z. Knight R. (2015). Dietary effects on human gut microbiome diversity. Br. J. Nutr. 113, S1–S5. 10.1017/S0007114514004127 PMC4405705 25498959

[B204] Yamaoka Y. Suehiro Y. Hashimoto S. Hoshida T. Fujimoto M. Watanabe M. (2018). Fusobacterium nucleatum as a prognostic marker of colorectal cancer in a Japanese population. J. gastroenterology 53 (4), 517–524. 10.1007/s00535-017-1382-6 28823057

[B205] Yan H. Bu P. (2021). Non-coding RNA in cancer. Essays Biochem. 65 (4), 625–639. 10.1042/EBC20200032 33860799 PMC8564738

[B206] Yang F. Xu Y. Liu C. Ma C. Zou S. Xu X. (2018). NF-κB/miR-223-3p/ARID1A axis is involved in *Helicobacter pylori* CagA-induced gastric carcinogenesis and progression. Cell Death Dis. 9 (1), 12. 10.1038/s41419-017-0020-9 29317648 PMC5849037

[B207] Yang L. Lu X. Nossa C. W. Francois F. Peek R. M. Pei Z. (2009). Inflammation and intestinal metaplasia of the distal esophagus are associated with alterations in the microbiome. Gastroenterology 137 (2), 588–597. 10.1053/j.gastro.2009.04.046 19394334 PMC2963147

[B208] Yang Y. Weng W. Peng J. Hong L. Yang L. Toiyama Y. (2017). Fusobacterium nucleatum increases proliferation of colorectal cancer cells and tumor development in mice by activating toll-like receptor 4 signaling to nuclear factor-κb, and up-regulating expression of MicroRNA-21. Gastroenterology 152 (4), 851–866. 10.1053/j.gastro.2016.11.018 27876571 PMC5555435

[B209] Yatsunenko T. Rey F. E. Manary M. J. Trehan I. Dominguez-Bello M. G. Contreras M. (2012). Human gut microbiome viewed across age and geography. Nature 486 (7402), 222–227. 10.1038/nature11053 22699611 PMC3376388

[B210] Yu J. Feng Q. Wong S. H. Zhang D. Liang Q. Y. Qin Y. (2017). Metagenomic analysis of faecal microbiome as a tool towards targeted non-invasive biomarkers for colorectal cancer. Gut 66 (1), 70–78. 10.1136/gutjnl-2015-309800 26408641

[B211] Yu T. Guo F. Yu Y. Sun T. Ma D. Han J. (2017). Fusobacterium nucleatum promotes chemoresistance to colorectal cancer by modulating autophagy. Cell 170 (3), 548–563. e16. 10.1016/j.cell.2017.07.008 28753429 PMC5767127

[B212] Yuan C. Burns M. B. Subramanian S. Blekhman R. (2018). Interaction between host MicroRNAs and the gut microbiota in colorectal cancer. mSystems 3 (3), e00205–17. 10.1128/mSystems.00205-17 29795787 PMC5954203

[B213] Yuan C. Steer C. J. Subramanian S. (2019). Host–MicroRNA–microbiota interactions in colorectal cancer. Genes 10, 270. 10.3390/genes10040270 30987065 PMC6523287

[B214] Zackular J. P. Rogers M. A. Mtt R. Schloss P. D. (2014). The human gut microbiome as a screening tool for colorectal cancer. Cancer Prev. Res. 7 (11), 1112–1121. 10.1158/1940-6207.CAPR-14-0129 PMC4221363 25104642

[B215] Zaidi A. H. Kelly L. A. Kreft R. E. Barlek M. Omstead A. N. Matsui D. (2016). Associations of microbiota and toll-like receptor signaling pathway in esophageal adenocarcinoma. BMC cancer 16 (1), 52. 10.1186/s12885-016-2093-8 26841926 PMC4739094

[B216] Zeller G. Tap J. Voigt A. Y. Sunagawa S. Kultima J. R. Costea P. I. (2014). Potential of fecal microbiota for early-stage detection of colorectal cancer. Mol. Syst. Biol. 10, 766. 10.15252/msb.20145645 25432777 PMC4299606

[B217] Zitvogel L. Ayyoub M. Routy B. Kroemer G. (2016). Microbiome and anticancer immunosurveillance. Cell 165 (2), 276–287. 10.1016/j.cell.2016.03.001 27058662

[B218] Zitvogel L. Daillere R. Roberti M. P. Routy B. Kroemer G. (2017). Anticancer effects of the microbiome and its products. Nat. Rev. Microbiol. 15 (8), 465–478. 10.1038/nrmicro.2017.44 28529325

[B219] Żółkiewicz J. Marzec A. Ruszczyński M. Feleszko W. (2020). Postbiotics-A step beyond pre- and probiotics. Nutrients 12 (8), 2189. 10.3390/nu12082189 32717965 PMC7468815

