# Correction: Understanding the role of the gut microbiome in gastrointestinal cancer: A review

**DOI:** 10.3389/fphar.2025.1662597

**Published:** 2025-07-30

**Authors:** Duygu Agagündüz, Ermelinda Cocozza, Özge Cemali, Ayşe Derya Bayazıt, Maria Francesca Nanì, Ida Cerqua, Floriana Morgillo, Suna Karadeniz Saygılı, Roberto Berni Canani, Paola Amero, Raffaele Capasso

**Affiliations:** ^1^ Department of Nutrition and Dietetics, Gazi University, Emek, Ankara, Türkiye; ^2^ MERCK S.P.A., Rome, Italy; ^3^ Department of Pharmacy, University of Naples “Federico II”, Naples, Italy; ^4^ Medical Oncology, Department of Precision Medicine, Università degli Studi della Campania “Luigi Vanvitelli”, Naples, Italy; ^5^ Department of Experimental Therapeutics, The University of Texas MD Anderson Cancer Center, Houston, TX, United States; ^6^ Department of Histology and Embryology, Kütahya Health Sciences University, Kütahya, Türkiye; ^7^ Department of Translational Medical Science and ImmunoNutritionLab at CEINGE Biotechnologies Research Center and Task Force for Microbiome Studies, University of Naples Federico II, Naples, Italy; ^8^ Department of Agricultural Sciences, University of Naples Federico II, Portici, Italy

**Keywords:** microbiome, gastrointestinal cancer, non-coding RNAs, therapeutics, diagnosis

In the published article, an incorrect **Funding** statement was provided. The corrected **Funding** statement appears below.

The original article has been updated.

